# Unusual behavior in the reactivity of 5-substituted-1*H*-tetrazoles in a resistively heated microreactor

**DOI:** 10.3762/bjoc.7.59

**Published:** 2011-04-21

**Authors:** Bernhard Gutmann, Toma N Glasnov, Tahseen Razzaq, Walter Goessler, Dominique M Roberge, C Oliver Kappe

**Affiliations:** 1 Christian Doppler Laboratory for Microwave Chemistry (CDLMC) and Institute of Chemistry, Karl-Franzens-University Graz, Heinrichstrasse 28, A-8010 Graz, Austria; 2 Institute of Chemistry, Analytical Chemistry, Karl-Franzens-University Graz, Universitätsplatz 1, A-8010 Graz, Austria; 3 Continuous Flow/Microreactor Technologies, Lonza AG, CH-3930 Visp, Switzerland

**Keywords:** flow chemistry, heterogeneous catalysis, microreactors, palladium, process intensification

## Abstract

The decomposition of 5-benzhydryl-1*H*-tetrazole in an *N*-methyl-2-pyrrolidone/acetic acid/water mixture was investigated under a variety of high-temperature reaction conditions. Employing a sealed Pyrex glass vial and batch microwave conditions at 240 °C, the tetrazole is comparatively stable and complete decomposition to diphenylmethane requires more than 8 h. Similar kinetic data were obtained in conductively heated flow devices with either stainless steel or Hastelloy coils in the same temperature region. In contrast, in a flow instrument that utilizes direct electric resistance heating of the reactor coil, tetrazole decomposition was dramatically accelerated with rate constants increased by two orders of magnitude. When 5-benzhydryl-1*H*-tetrazole was exposed to 220 °C in this type of flow reactor, decomposition to diphenylmethane was complete within 10 min. The mechanism and kinetic parameters of tetrazole decomposition under a variety of reaction conditions were investigated. A number of possible explanations for these highly unusual rate accelerations are presented. In addition, general aspects of reactor degradation, corrosion and contamination effects of importance to continuous flow chemistry are discussed.

## Introduction

Microreactor technology has opened up new avenues for synthetic organic chemistry [1–6] and the chemical manufacturing industry [7–8]. Traditionally, most synthetic transformations performed in microreactors have involved ambient or even low-temperature conditions in order to conduct highly exothermic reactions safely [1–9]. More recently, following the concepts of “Process Intensification” and “Novel Process Windows” [10–12], flow chemistry executed in high-temperature and/or high-pressure regimes have become increasingly popular [13]. High-temperature processing offers many distinct advantages as demonstrated by the recent success of microwave-assisted organic synthesis [14–18]. In microwave chemistry, reaction times can often be reduced from hours to minutes by efficient and rapid direct dielectric heating of the reaction mixture in a sealed vessel to temperatures far above the boiling point of the solvent under atmospheric conditions. Since batch microwave chemistry is inherently difficult to scale up to production quantities [14–16], translating high-speed, high-temperature microwave chemistry to scalable continuous flow processes is becoming increasingly important. In the past few years our research group [19–25] has reported a number of successful case studies where initial reaction optimization for a variety of synthetic transformations was performed under batch microwave conditions, followed by translation to high-temperature/high-pressure scalable continuous flow processes (“microwave-to-flow” paradigm) [26–28].

A recent example involves the synthesis of 5-substituted-1*H*-tetrazoles via an azide–nitrile cycloaddition pathway, using sodium azide (NaN_3_) as an inexpensive azide source and acetic acid (AcOH) as the reagent/catalyst in a NMP/H_2_O solvent mixture [25]. These 1,3-dipolar cycloadditions which involve in situ generated free hydrazoic acid (HN_3_) were performed on small scale (≈1 mL) at 220 °C in a microwave batch reactor with reaction times of 4–15 min, depending on the reactivity of the nitrile [25]. Despite the comparatively high reaction temperatures, virtually no side products were observed in these transformations and the desired tetrazole products were isolated in high yield and purity after a simple work-up procedure [25]. Since HN_3_ is not only extremely toxic but also highly explosive, a scale-up of this batch protocol is clearly not possible. A key advantage of using microreactors compared to conventional batch reactors is the ability to process potentially hazardous compounds or reagents safely [1–9,29–32]. In a continuous flow system, synthetic intermediates can be generated and consumed in situ, which eliminates the need to store toxic, reactive or explosive intermediates and thus makes the synthetic protocol safer. However, initial attempts to convert the azide–nitrile cycloaddition protocols to a high-temperature continuous flow process using a stainless steel microtubular flow reactor have failed. Instead of the desired tetrazole products, formation of a number of decomposition products was observed.

In this paper the mechanistic details and kinetic profiles of high-temperature tetrazole decompositions in both microwave batch and metal-based microreactors are investigated. A number of possible explanations for the unusual failure to convert batch to flow conditions for this specific transformation are presented. In addition, often ignored aspects of reactor degradation, corrosion and contamination effects in flow chemistry are also discussed.

## Results and Discussion

### Flow degradation of 5-benzhydryl-1*H*-tetrazole

**Scheme 1:**
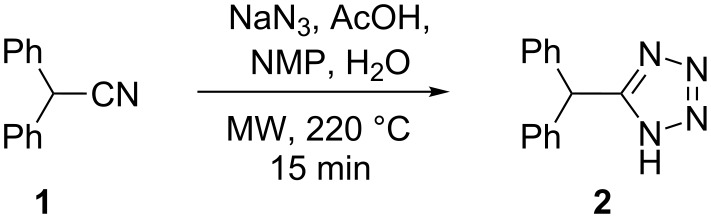
Azide–nitrile cycloaddition under batch microwave conditions.

As a model system for tetrazole formation the microwave-assisted cycloaddition of diphenylacetonitrile (**1**) with NaN_3_ was studied (Scheme 1). After considerable experimentation [25] an optimum set of conditions that fulfilled both the requirement of reaction homogeneity and reaction rate whilst at the same time providing clean and complete nitrile to tetrazole conversion involved the use of NMP as solvent, AcOH as Brønsted acid and H_2_O as co-solvent. Thus, 2.5 equiv of NaN_3_ and a 5:3:2 ratio of NMP/AcOH/H_2_O at 220 °C (≈15 bar) led to full conversion of the reactants to the desired 5-benzhydryl-1*H*-tetrazole (**2**) at a 0.69 M nitrile concentration within 15 min, and furnished the product in 81% isolated yield.

**Figure 1:**
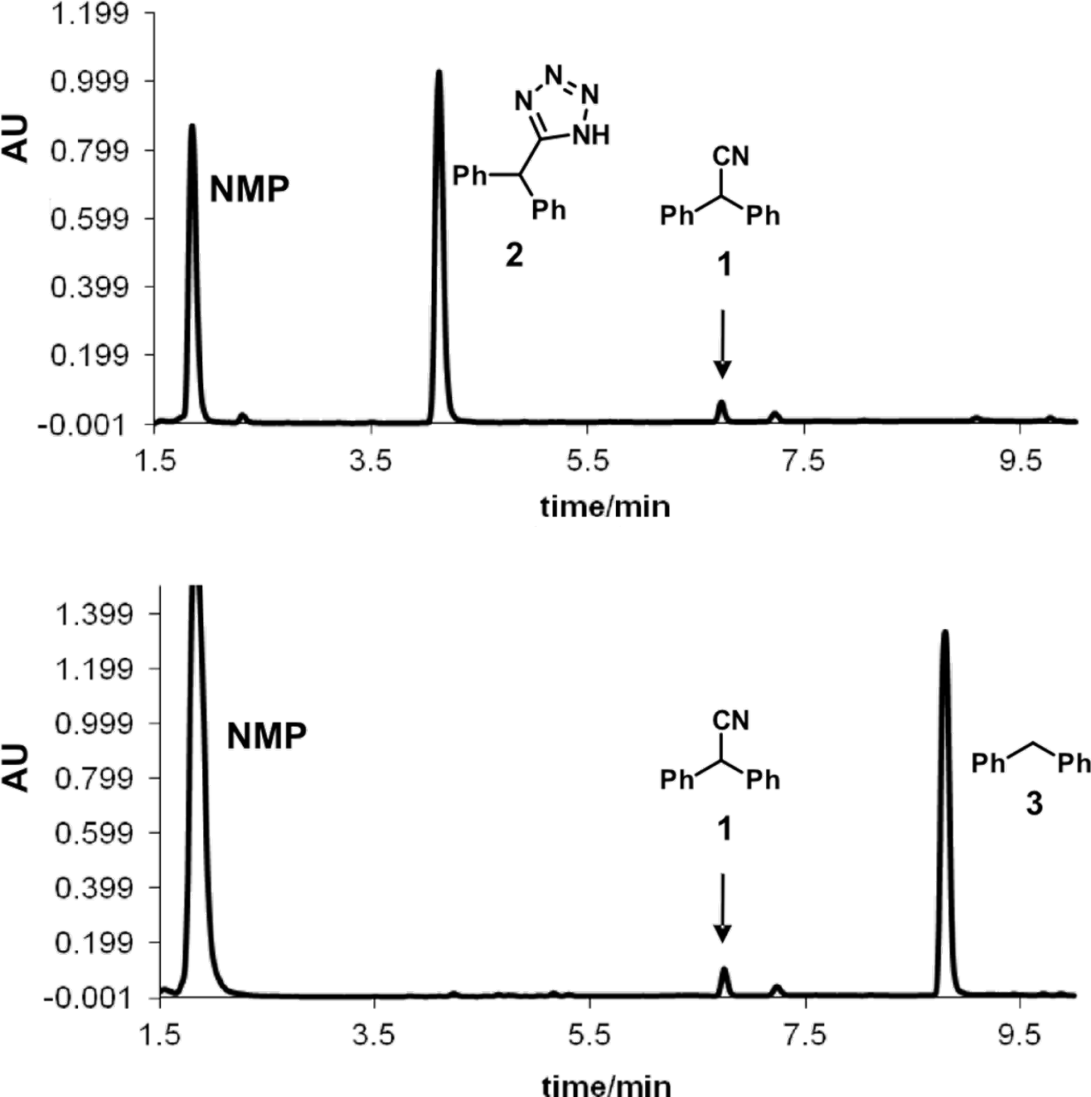
HPLC-UV chromatograms (215 nm) of crude reaction mixtures from the cycloaddition of diphenylacetonitrile (**1**) with NaN_3_ (Scheme 1) comparing microwave batch (top) and continuous flow conditions (bottom). Reaction parameters: 220 °C, ≈16 min, 2.5 equiv NaN_3_, NMP/AcOH/H_2_O 5:3:2.

To our surprise, however, we were initially unable to translate these high-temperature batch conditions obtained in sealed Pyrex glass reaction vessel (using either conventional or microwave heating) [25] to a continuous flow format. The microreactor system used for these studies was a high-temperature, high-pressure microtubular flow unit that can be used for processing homogeneous reaction mixtures [24]. This reactor uses stainless steel coils of variable length (4, 8 or 16 mL internal volume) that can be directly heated across their full length (5–20 m) by electric resistance heating to temperatures up to 350 °C. Thermocouples are attached to the outer surface of the stainless steel tubing at two different points along the length of the coil to measure the temperature of the coils. In the actual cycloaddition experiment, the reaction mixture was introduced to the reactor block containing a 8 mL stainless steel coil (SX 316L, i.d. 1 mm) heated to 220 °C via a standard HPLC pump at a flow rate of 0.5 mL/min. This translates to a residence time of 16 min inside the heated coil, comparable to the reaction time in the batch microwave experiment (Scheme 1). However, instead of the anticipated tetrazole **2** the only major product observed by HPLC-UV monitoring was diphenylmethane (**3**) (Figure 1). This very unusual behavior was in stark contrast to our previous experience in converting microwave to flow conditions with the same instrument [19–24], and was not limited to nitrile **1** but was also observed for other nitrile building blocks.

We initially speculated that the observed disintegration of the tetrazole nucleus could somehow be connected to the metal surface of the steel reactor. The particularly high surface-to-volume ratio in a micro structured reactor (the inner surface area of a 16 mL coil with an inner diameter of 1 mm is 640 cm^2^) may entail pronounced reactor wall effects and unexpected/undesired side and degradative reactions [33]. The stainless steel reactor contains a variety of metals and the reactor walls could possibly act as heterogeneous catalysts (surface catalysis) [34]. Furthermore, the surface can potentially be considered as a metal oxide which usually forms surface hydroxyl groups in contact with water. Depending on the pH of the processed mixture, the surface hydroxides are protonated or deprotonated and, hence, the surface is uncharged at the isoelectric point or positively (negatively) charged at lower (higher) pH values [35]. In this regard, the surface area, the material of construction and the reactor use history have to be considered. In addition, the stainless steel components of the flow reactor are prone to corrosion if harsh conditions, such as concentrated acids are used [36]. Hydrazoic acid itself (p*K*_a_ 4.7) is both a strong oxidant (*E*° = 1.96 V) and, in the presence of oxidants, a strong reducing agent (*E*° = −3.09V). It also dissolves metals such as Fe, Zn, Mn and Cu. Thus, HN_3_ should in principle be considered as incompatible with the use of steel reactors [37]. Furthermore, iron nitrides are readily formed from HN_3_ and Fe metal at temperatures around 100 °C which represents a serious safety hazard [38].

On the other hand, it has been reported that the decomposition of tetrazoles can be catalyzed by a whole range of metals. For example, Cu powder was found to lower the decomposition temperature of 1,5-diphenyltetrazole by about 60 °C [39]. However, in case of the parent tetrazole (CN_4_H_2_) itself, differential scanning calorimetry (DSC) experiments suggest that the decomposition onset temperature does not change significantly when the material is contaminated with either Fe or 316 stainless steel [40].

Whatever the reason for the inability to translate microwave batch conditions optimized in Pyrex glass to a continuous flow regime with a stainless steel coil, the problem was ultimately solved by employing a passivated silica coated stainless steel coil (Sulfinert^®^) mimicking a glass environment [41], in combination with the use of a flow reactor that employed a standard Al heating block as a coil heater [25]. Using this set-up, a general and scalable method for the continuous flow synthesis of 5-substituted-1*H*-tetrazoles via the addition of HN_3_ to organic nitriles was developed [25]. For specific substrates the coil temperature could be raised up to 260 °C (2.5 min residence time) without any significant amounts of decomposition products being detected in the crude reaction mixture [25].

**Figure 2:**
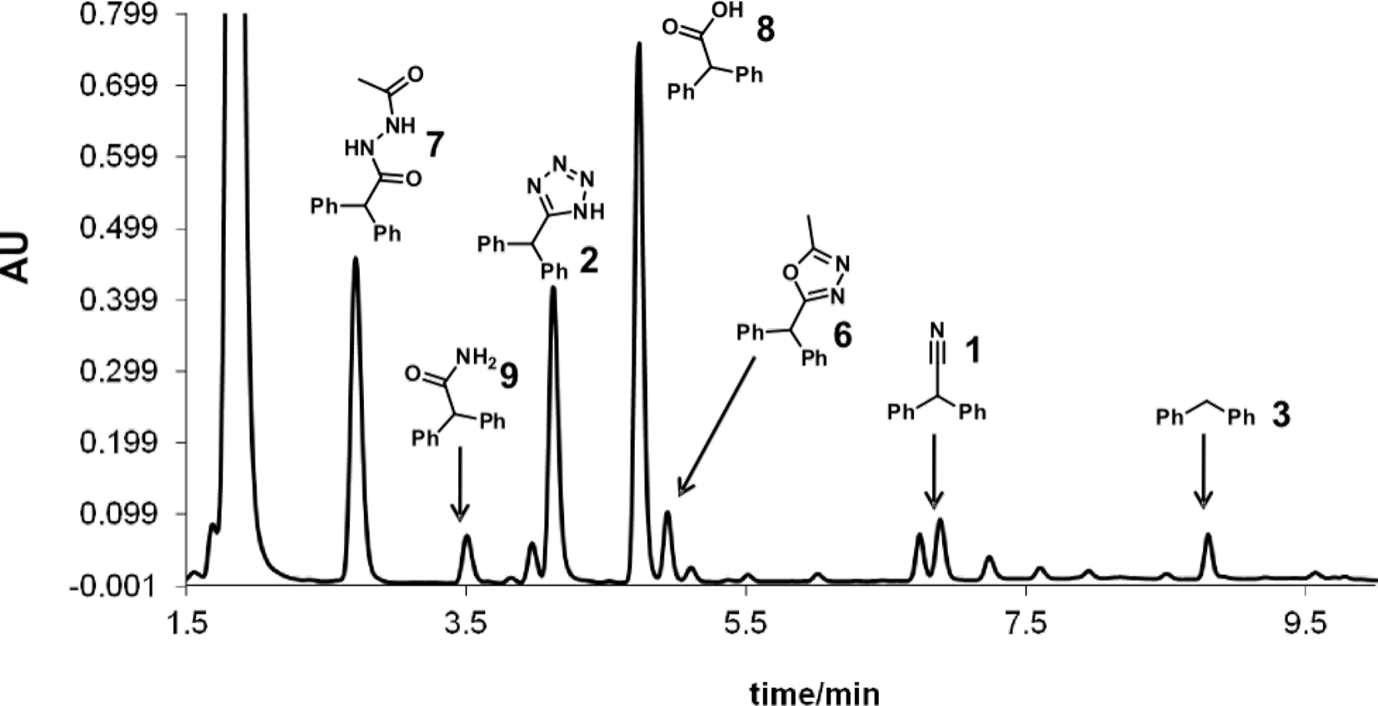
HPLC-UV chromatogram (215 nm) showing the decomposition of tetrazole **2** in NMP/AcOH/H_2_O 5:3:2 (0.125 M) after heating in a stainless steel flow coil at 180 °C for 5.3 min.

Control experiments subjecting the isolated pure tetrazole **2** to a NMP/AcOH/H_2_O solvent mixture quickly revealed that indeed the very low stability of the tetrazole nucleus in the resistively heated stainless steel flow reactor was responsible for the observed decomposition of the product. The pure tetrazole **2** in NMP/AcOH/H_2_O (5:3:2) started to decompose at temperatures as low as 150 °C after a only few min residence time under flow conditions and the HPLC-UV traces of the crude reaction mixtures became fairly complicated (Figure 2). On increasing the reactor temperature or applying longer residence times, however, all peaks except the diphenylmethane (**3**) signal vanished. At 220 °C, the temperature contemplated for the tetrazole synthesis, diphenylmethane (**3**) was the only detectable product after ≈10 min of residence time.

**Scheme 2:**
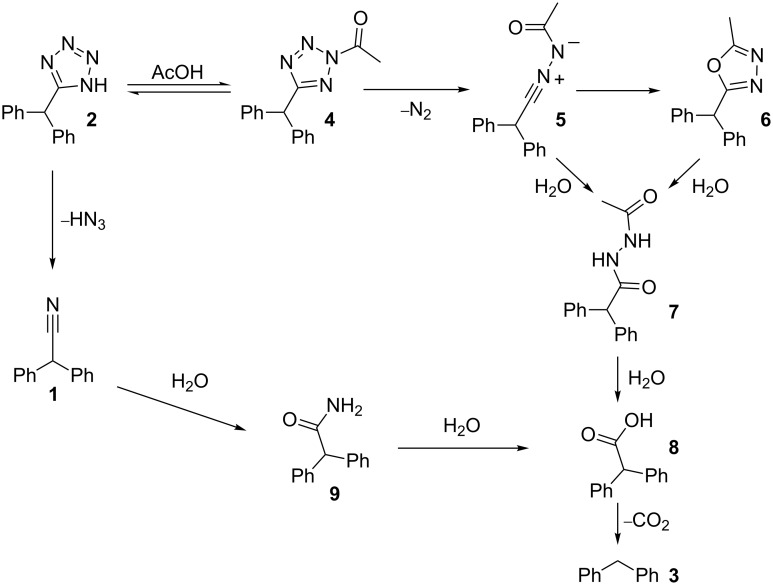
Possible decomposition mechanisms for tetrazole **2** in NMP/AcOH/H_2_O.

Apparently, the degradation of tetrazole **2** in the NMP/AcOH/H_2_O solvent mixture involves various consecutive reactions and/or parallel pathways that finally channel to diphenylmethane (**3**). We have identified virtually all the peaks in the HPLC chromatograms and therefore have a rather complete picture of the complex degradation processes (Scheme 2, Figure S1 in Supporting Information File 1). The main degradation path starts with the *N*-acetylation of the tetrazole nucleus at position 2. The resulting *N*-acetyltetrazole **4** looses nitrogen to form nitrilimine **5**. Interception of the nitrilimine dipolar intermediate by water produces *N’*-acetyl-diphenylacetohydrazide (**7**) (the first detectable intermediate in this sequence), while intramolecular interception leads to the oxadiazole **6**. This mechanism for the degradative acylation of tetrazoles as shown in Scheme 2 was suggested by Huisgen and coworkers in 1958 [42–43]. An alternative mechanism, whereby the 5-substituted-1*H*-tetrazole is acetylated at position N1 followed by ring opening at the 1,2-position, elimination of nitrogen from the resulting azido group and a subsequent 1,2-migration of the acylimido group from carbon to nitrogen to give the same nitrilimine intermediate was ruled out by Herbst via ^15^N labeling studies [44]. The degradative acylation of 5-substituted-1*H*-tetrazoles with acyl halides is in fact an elegant method for the synthesis of 1,3,4-oxadiazoles [45–46]. In the NMP/AcOH/H_2_O solvent mixture, however, the formation of the oxadiazole **6** can scarcely compete with the intermolecular addition of water and oxadiazole **6** was therefore detectable only in minor amounts. Using NMP/AcOH as the solvent system, 2-benzhydryl-5-methyl-1,3,4-oxadiazole (**6**) became one of the major products in the flow reactor under these reaction conditions. The resulting hydrazide **7** can be expected to have a weak N–N bond, however, the products obtained from the decomposition of diacylhydrazides are generally considered to arise via polar pathways rather than via a radical path [47]. Under the employed reaction conditions the hydrazide apparently hydrolyzes to diphenylacetic acid (**8**) which finally decarboxylates to yield diphenylmethane (**3**). Decarboxylation may proceed via an intramolecular, concerted mechanism as proposed for β,γ-unsaturated acids [48].

In addition to these structures, a different set of intermediates resulting from a second decomposition pathway were identified, although in much smaller quantities. The second path possibly involves cyclo-reversion of the tetrazole to the nitrile **1**, hydrolysis of the nitrile to the amide **9** and then further to the carboxylic acid **8**, which again finally decarboxylates to produce diphenylmethane (**3**) (Scheme 2) [49]. Further details and control experiments supporting the proposed decomposition pathways are discussed in the Supporting Information File 1.

**Scheme 3:**
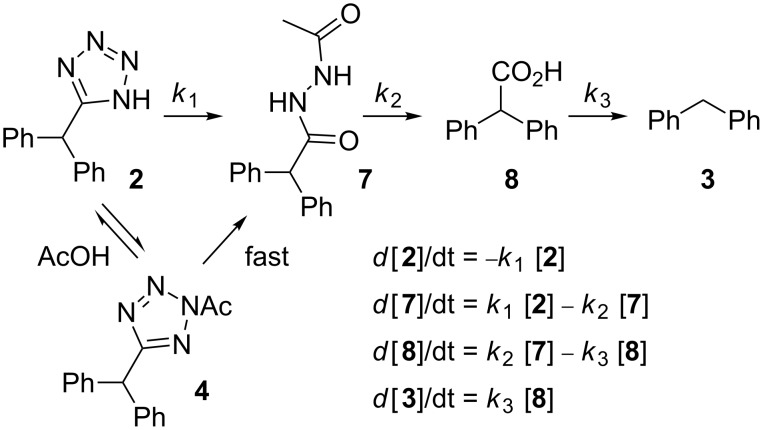
Reaction steps for the degradation of tetrazole **2** and the corresponding rate equations.

**Figure 3:**
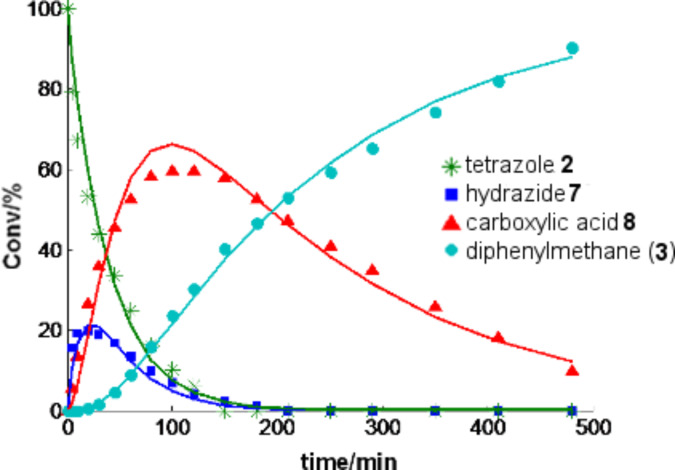
Decomposition of tetrazole **2** at 240 °C in a NMP/AcOH/H_2_O 5:3:2 mixture (0.125 M) (points: experimental results; solid lines: predicted with a rate law according Scheme 3 with *k*_1_ = 1.11 × 10^−3^, *k*_2_ = 0.432 × 10^−3^ and *k*_3_ = 0.0832 × 10^−3^ s^−1^). Conversions in percent were derived from HPLC-UV (215 nm) peak area integration without correction for response factors. The corresponding conversions for 250 °C and 260 °C and additional data are shown in the Supporting Information File 1 (Figures S2–S4).

Notably, exactly the same decomposition pathway involving the identical set of intermediates as in the stainless steel reactor were indeed also observed in a microwave batch reactor using a Pyrex glass vessel, but the reactions were nowhere near as fast. In the microwave reactor equipped with an accurate internal fiber-optic temperature probe [50] there was no appreciable disintegration of tetrazole **2** in the NMP/AcOH/H_2_O system at 220 °C after 30 min. In order to obtain reasonable decomposition rates for a kinetic analysis, the reaction temperature had to be increased to 240 °C, but even at 240 °C the decomposition process required many hours. The intermediates from the “second path” (Scheme 2) were hardly detectable and the resulting experimental data could be fitted nicely with the rate law shown in Scheme 3 with each step assumed to be (pseudo) first order. A least square fit revealed *k*_1_ = 1.11 × 10^−3^, *k*_2_ = 0.432 × 10^−3^ and *k*_3_ = 0.0832 × 10^−3^ s^−1^ at 240 °C (Figure 3). Control experiments in a microwave reaction vial constructed from strongly microwave absorbing silicon carbide (SiC), which shields the vessel contents from the electromagnetic field and therefore mimics a conventionally heated autoclave experiment, provided identical results and demonstrates that the observed decomposition in the microwave reactor was the result of purely thermal effects [51–52].

**Figure 4:**
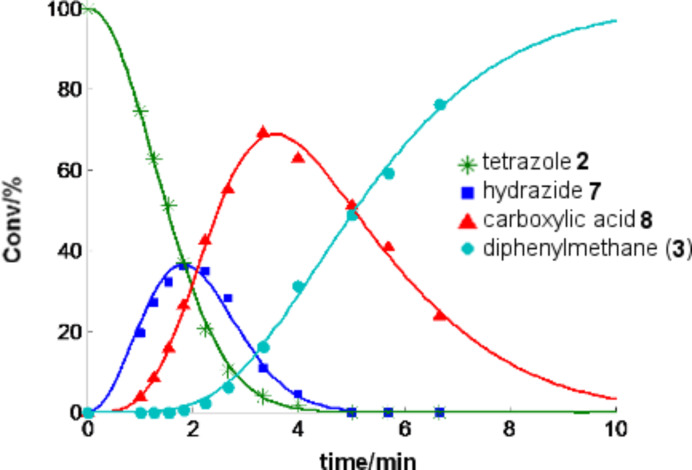
Decomposition of tetrazole **2** in a 4 mL resistance heated stainless steel coil at a nominal temperature of 220 °C in a NMP/AcOH/H_2_O 5:3:2 mixture (0.125 M) (points: experimental results; solid lines: predicted with a rate law according Scheme 3 on the assumption that the rate constants are proportional to the residence time *k*_i_ = *a*_i_ · *t* (i.e., inversely proportional to the flow rate *k*_i_ = *a*_i_ · *V* ∙ *v*^−1^, *t* = residence time, *V* = reactor volume, *v* = flow rate) with *a*_1_ = 0.169 × 10^−3^, *a*_2_ = 0.166 × 10^−3^ and *a*_3_ = 0.0207 × 10^−3^ s^−2^). Conversions in percent were derived from HPLC-UV (215 nm) peak area integration without correction for response factors. Similar curves for different coils are shown in Figure S5 in the Supporting Information File 1.

A comparison of the batch and flow data presented above shows that the tetrazole decomposition in the stainless steel flow coil is ≈100 times faster than decomposition in the Pyrex vial at the same temperature. The rate constants for the decomposition process in the resistively heated flow reactor are apparently not simple first order and appear to depend on the flow rate/residence time and show decreasing rate constants with increasing flow rates/decreasing residence times. For example, the experimental data obtained in a 4 mL stainless steel coil can be fitted nicely with the rate law shown in Scheme 3 assuming that the rate constants are inversely proportional to the flow rate (*k* ~ v^−1^) (Figure 4). Although some differences in the rate of decomposition between individual coils (4, 8, 16 mL internal volume, different age and history) were noticeable (Figure S5, Supporting Information File 1), in all cases the disintegration of the tetrazoles was dramatically faster compared to the microwave batch conditions.

In order to investigate if the enhanced tetrazole decomposition in the stainless steel coils is connected to metal contamination as a result of steel corrosion phenomena, a series of inductively coupled plasma mass spectrometry (ICPMS) experiments were performed. ICPMS analysis of a NMP/AcOH/H_2_O (5:3:2) mixture pumped through the continuous flow reactor at 220 °C (residence time 5 min) revealed that a range of metals are released from the coil under these conditions. Especially Fe (up to 113 ± 6 mg/L) but also Ni (up to 21.0 ± 1.0 mg/L), Cr (up to 32.3 ± 1.5 mg/L) and Mn (up to 4.9 ± 0.3 mg/L) were found in the processed solvent (Table S1, Supporting Information File 1). We hypothesized that several of these metals liberated from the stainless steel capillary may catalyze some of the decomposition steps in solution. Indeed, for example, deliberately added stoichiometric amounts of Fe_2_O_3_ markedly accelerated the decarboxylation of diphenylacetic acid (**8**) under batch microwave conditions, so that the acid no longer accumulated. The other degradation steps, however, remained virtually unaffected (Figure S6, Supporting Information File 1).

The detected individual amounts of metals in the solvent mixture after treatment in coils of different length varied significantly and correlated somewhat with the age and history of the coils. The decomposition rates, on the other hand, did not show a correlation with the detected quantity of any of the released metals (Figure S5, Supporting Information File 1). Furthermore, decomposition of tetrazole **2** under batch microwave conditions in Pyrex using a NMP/AcOH/H_2_O mixture pre-treated as described in the stainless steel reactor was not appreciably faster compared to the decomposition in “fresh” NMP/AcOH/H_2_O (Figure S7, Supporting Information File 1). It thus appears that the surprisingly fast degradation of tetrazole **2** in the resistively heated steel reactor compared to the microwave batch experiment at the same temperature is not the result of homogenous catalysis by some of the released metals.

In order to obtain further insights into this remarkable enhancement of tetrazole decomposition, the same reaction was subsequently carried out in flow devices using metal coils made from different materials heated either in an oil bath or with an Al heating block. Remarkably, the stability of tetrazole **2** in a 5.21 mL Hastelloy C-4 coil (i.d. 2.0 mm) at 240 °C was very close to the stability observed in the microwave batch experiment (Figure S8, Supporting Information File 1). The superior resistance of the Hastelloy material toward the NMP/AcOH/H_2_O mixture at high temperature was also evident from an ICPMS analysis of the solvent mixture processed in the Hastelloy C-4 capillary at 220 °C (5 min residence time, 1.0 mL/min flow rate). Hastelloy is a high-performance Ni-Cr-Mo alloy with enhanced corrosion stability compared to standard steel materials under high temperature conditions [53]. Therefore, only comparatively small amounts of released Ni (5.3 ± 1.1 mg/L), W (2.4 ± 0.6 mg/L) and Mo (2.0 ± 0.4 mg/L) were detected in the processed solvent mixture. The amounts of other metals, including Fe, were negligible compared to the background values (Table S1, Supporting Information File 1).

In an additional experiment, we demonstrated that the decomposition of the pure tetrazole **2** in a stainless steel capillary heated on an Al heating block was only marginally faster than the decomposition in the microwave reactor at the same measured reaction temperature. A least square fit of the obtained experimental data with the kinetic model depicted in Scheme 3 gave *k*_1_ = 2.52 × 10^−3^, *k*_2_ = 1.15 × 10^−3^ and *k*_3_ = 0.184 × 10^−3^ s^−1^. All reaction steps are hence about two times faster than under microwave conditions (Figure S9, Supporting Information File 1). This rather small difference in the decomposition rate (compared to the factor 100 found in the resistively heated coils) can be explained by an inaccurate temperature calibration of the Al block coil heating system used for heating the stainless steel capillary. If metal/surface catalysis would be involved it could be expected that the individual reaction steps of the decomposition pathway would be affected to different extents. However, the rate constants for all consecutive reactions in the flow reactor at a measured temperature of 240 °C were very close to those found under microwave batch conditions at 250 °C (Figure S10, Supporting Information File 1). These results strongly suggest that the degradation process for tetrazoles of type **2** is indeed not influenced by the stainless steel material and thus does not involve heterogeneous/surface catalysis phenomena.

Ultimate proof that the enhanced tetrazole decomposition in the resistively heated coils is related to the method of heating and not to the coil material itself was obtained from a control experiment where the very same 8 mL stainless steel coil initially used in the dedicated flow reactor employing electric resistance heating [24] was subsequently used in a conductively heated experiment. This was achieved by immersing the complete reactor block containing the steel coil and accessories into a well agitated silicone oil bath. Remarkably, with a 10 min residence time for both types of flow experiments, the conductively heated flow run at 192 °C showed no sign of tetrazole decomposition, whilst with electric resistance heating, complete disintegration of the tetrazole occurred at even lower temperatures (Figure S11, Supporting Information File 1).

At the moment we have no compelling explanation for the dramatic discrepancies observed in tetrazole decomposition rates comparing different heating principles. One possible reason for the exceptionally fast disintegration of the tetrazoles in flow reactors utilizing direct electrical resistance heating would be the occurrence of extreme hot spots or uneven temperature distributions along the capillary. To explain the observed degradation rates, however, average temperatures well above 300 °C in the resistively heated reactor have to be assumed (Figure S10, Supporting Information File 1). This appears unlikely taking our previous experience in microwave-to-flow translations into account, where the same instrument and coils were used, but inconsistencies as seen in the tetrazole decomposition have never been observed [19,24]. As the experiments in the resistively heated flow reactor were typically performed at a higher pressure (140 bar) than the corresponding experiments in a conductively heated flow instrument (34 bar), the influence of reaction pressure on tetrazole decomposition was also investigated. Although some differences could be observed performing flow decomposition experiments at 50 and 140 bar in the resistively heated flow reactor, respectively (Figure S12, Supporting Information File 1), these differences were not large enough to suggest a genuine pressure effect. Finally, the possibility of electrochemical phenomena, as a result of the electric current passing through the steel coil interacting with the reaction mixture, were considered (Figure S13, Supporting Information File 1) [54]. Comparative experiments for the tetrazole decomposition carried out in batch mode at 220 °C for 10 min in a short stainless steel loop (≈300 µL) heated either by direct electrical resistance heating with a DC-switching power supply (Figure S14, Supporting Information File 1), or by immersion into an oil bath, support the notion that the enhanced decomposition is somehow related to the electric current. While the batch experiment in the conductively heated coil showed little to no tetrazole decomposition, diphenylmethane (**3**) and diphenylacetic acid (**8**) were the main products in the resistively heated run (Figure S15, Supporting Information File 1).

### Corrosion effects influencing flow chemistry in steel reactors

Extreme care is required in the selection of the reactor materials when solutions containing even relatively dilute concentrations of HCl or other Brønsted acids are handled in a flow environment [36]. The corrosion results described earlier involving comparatively benign mixtures of NMP/AcOH/H_2_O (5:3:2) passed through stainless steel coils at 220 °C provide testimony to the fact that even mild acids (the p*K*_a_ of AcOH is 4.75) can act as corrosive reagents on stainless steel in an elevated temperature regime. Aqueous HCl is a much stronger acid and lacks the oxidizing properties that stainless steel requires to maintain its “passive” corrosion resistant surface layer [55]. Furthermore, chloride containing acidic solutions will in many situations show a corrosive nature similar to HCl itself. The corrosion attack of HCl, as with most acids, is highly dependent on the temperature but all common stainless steel types should be considered non-resistant to HCl at any concentration and temperature [36]. Hastelloy type reactor materials in turn are known to offer better corrosion resistance in both reducing and oxidizing environments [53].

In order to evaluate the relative corrosiveness of HCl at different concentrations and in different reactor environments, a test reaction was developed that can readily reflect the level of corrosion. For this purpose a 0.5 M solution of nitrobenzene (**10**) in EtOH containing varying amounts of aqueous conc. HCl (0–1.0 M) was flowed through reactor coils made out of PTFE, SX 316L stainless steel or Hastelloy C-4 at 150 °C (≈20 min residence time) (Table 1). The “nascent” hydrogen formed in the corrosion process causes unwanted reduction reactions when susceptible groups, e.g., nitro groups, are in the molecule. The reduction of nitro compounds with Fe in the presence of HCl is known as the Bechamp reduction and large amounts of aniline from nitrobenzene are produced by this reaction [56]. As expected, the reduction of nitrobenzene (**10**) to aniline (**11**) in metal coils increased steadily with increasing HCl concentration. With R–NO_2_ + 6 H^+^ + 6 e^−^ → R–NH_2_ + 2 H_2_O, the reactions were more or less quantitative with respect to HCl after 20 min at 150 °C in a stainless steel coil. Significant reduction to aniline was, however, also observed in a Hastelloy coil under these conditions. As expected, no reduction was experienced in reactor coils made out of chemically inert PTFE, which unfortunately lacks the temperature and pressure resistance to perform genuine high-temperature/high-pressure flow chemistry (the autogenic pressure in the Bechamp reduction at 150 °C was ≈9 bar) [13]. The corrosion in steel and Hastelloy coils was also clearly evident as strongly colored solutions, probably due to dissolved Fe^II/III^, exited the coil (Figure S16, Supporting Information File 1).

**Table 1:** Conversion of nitrobenzene (**10**) to aniline (**11**) in different flow environments.^a^

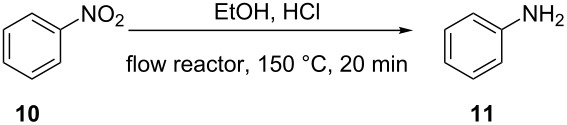			

HCl equiv^b^	Conversion (%)^c^		

	Stainless steel	Hastelloy	PTFE

0.0	0	0	0
0.5	5	2	0
1.0	14	7	0
2.0	31	19	0

^a^Reaction conditions: conductively heated (150 °C, 20 min) stainless steel (20 mL, flow rate 1.0 mL/min), Hastelloy (5.21 mL, flow rate 0.26 mL/min) and high-temperature PTFE (14 mL, flow rate 0.7 mL/min) coils. ^b^HCl equiv with respect to nitrobenzene (**10**). ^c^Conversions were obtained by GC-FID monitoring.

### Reactor contamination by catalytically active transition metals

Another critical issue in flow chemistry that is often overlooked is reactor contamination (fouling) as a result of substrate, solvent, reagent or catalyst degradation inside the flow device. In high-temperature flow chemistry, the problem is particularly serious as the sometimes rather extreme temperatures on the reactor walls may lead to the decomposition of otherwise quite stable materials. The potential disintegration may be further enhanced by material and/or surface phenomena resulting from the high surface-to-volume ratio in a micro-reactor environment. Since, in most cases, microreactors or related flow devices (coils, tubular reactors) for high-temperature/high-pressure applications are made out of non-transparent materials (i.e., stainless steel, alloys, ceramics) [13], the problem is further aggravated since reactor contamination may not be immediately obvious. As flow devices are generally designed for long-term use, the inadvertent accumulation of chemical contaminants inside of these reactors must always be taken into account when interpreting the results from flow chemistry experiments. In the case of the tetrazole decomposition discussed above (Scheme 2), a careful inspection of the individual kinetic profiles obtained in stainless steel coils that had been exposed to different chemistries over several months of usage (Figure 4 and Figure S5 in Supporting Information File 1) clearly reveals that the age/history of the reactor can have an influence on the chemical transformations occurring inside these flow devices.

**Scheme 4:**
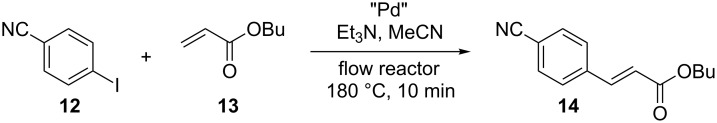
Mizoroki–Heck coupling under continuous flow conditions.

The use of transition metal catalysts for carbon–carbon or carbon–heteroatom bond formation under continuous flow conditions represents an interesting opportunity for studying the effects of metal contaminations inside of microreactors, as many of these coupling reactions proceed in the presence of extremely small quantities of transition metal catalysts in so-called “homoeopathic” doses [57–58]. In a recent publication we reported the Mizoroki–Heck coupling of 4-iodobenzonitrile (**12**) with *n*-butyl acrylate (**13**) under high-temperature continuous flow conditions (150–190 °C) employing Pd(OAc)_2_ as a homogeneous pre-catalyst and a stainless steel-based coil flow system (Scheme 4) [59]. With only 0.01 mol % of Pd(OAc)_2_ as catalyst, MeCN as solvent and triethylamine as base gave cinnamic ester **14** in very high yield (≈95%) and good selectivity. It can be assumed that at the comparatively high reaction temperatures applied in the high-temperature flow reactor, the homogeneous Pd pre-catalyst will be rapidly converted into Pd colloids/nanoparticles with high catalytic activity [57–58].

In the course of these investigations, we noticed that catalytically active Pd species were apparently retained inside the steel reactor coil on performing these Pd-catalyzed high-temperature coupling transformations, despite an extensive washing regime with pure solvent. This became very obvious, since running the Mizoroki–Heck coupling shown in Scheme 4 without any added Pd catalyst still led to complete conversion in subsequent flow experiments utilizing the same coil. Control experiments in batch mode confirmed that the untreated steel material itself (SX 316L) cannot catalyze Mizoroki–Heck couplings of this type, despite of the fact that Ni and even Fe-catalyzed carbon–carbon bond forming reactions are well known in the literature [60–61]. Furthermore, very low levels of conversion were obtained in this coupling when using a pristine, unused stainless steel coil in the flow reactor without the addition of a Pd catalyst.

In order to investigate these phenomena in more detail, a new set of experiments involving “palladated” steel coils was designed. For this purpose, a 16 mL internal volume stainless steel coil (≈20 m of 1.0 mm i.d. coil) was “loaded” with Pd by processing ≈2 mL of the Mizoroki–Heck reaction mixture through the coil under reaction conditions (180 °C, 1.6 mL/min flow rate) with 1 mol % of the Pd(OAc)_2_ as pre-catalyst. As expected, the desired product **14** was obtained in 94% isolated yield after chromatographic purification. To clean the instrument extensive washing at 170 °C for 20 min with MeCN was performed. The so conditioned steel coil was then used for processing a new portion of the reaction mixture that did not contain any Pd catalyst. Remarkably, analysis of the reaction mixture by GC-MS demonstrated full conversion to cinnamic ester **14** in the initial product fractions exiting the flow reactor. To establish for how long this “palladated” steel coil could be used without additional amounts of catalyst being added, 15 mL of reaction mixture containing 1.125 g of aryl iodide **12** (8.45 mmol) were processed through the coil under the same reaction conditions (180 °C, 1.6 mL/min flow rate). The analytical results clearly demonstrated that after processing ≈7 mL of reaction mixture conversions started to decrease and ultimately reached almost 0% after 12 mL.

With the purpose of investigating the nature of the Pd leaching from the coil in more detail a freshly “palladated” coil was subjected to a similar series of experiments, measuring the amount of leached Pd by ICPMS analysis (Table 2). While the background leaching for pure solvent at room temperature and at the reaction temperature was relatively low (Table 2, entries 1 and 2), significantly higher levels of Pd were found in samples derived from the Mizoroki–Heck reaction mixture (Table 2, entry 3). These results, both in terms of decreasing conversion and Pd leaching are therefore analogous to the experiments using Pd/C as a heterogeneous pre-catalyst in continuous flow Mizoroki–Heck chemistry [59]. Catalysis essentially proceeds via dissolution/re-adsorption of Pd from the support (here from the steel coil). The mechanism is quasi-homogeneous with small Pd^0^ species (colloids/nanoparticles) in solution acting as the catalytically active species. Presumably, the heterogeneous “Pd-on-steel” pre-catalyst is initially solubilized by oxidative addition of the aryl iodide and enters the catalytic cycle in the form of a soluble Pd species. Therefore, significant levels of Pd leaching are observed for the reaction mixture, not for pure solvent [59].

**Table 2:** Leaching of Pd from a “palladated” steel coil for the Mizoroki–Heck coupling of aryl iodide **12** with *n*-butyl acrylate (**13**) (Scheme 4).^a^

Entry	Reaction mixture composition	Leaching [μg Pd]^b^

1	MeCN at rt	4.9
2	MeCN at 180 °C	10.9
3	Reaction mixture at 180 °C	52.9

^a^Complete reaction mixture composition: 0.65 mmol aryl iodide **12**, 1.5 equiv *n*-butyl acrylate (**13**), 1.5 equiv Et_3_N, 2 mL MeCN. Conditions: 16 mL stainless steel coil, 180 °C, 1.6 mL/min flow rate. ^b^Determined by ICPMS analysis of the product contained in a 10 mL fraction.

We suspect that the Pd metal inside the steel coil is present in the form of a thin film of nanometer-sized Pd crystallites, very similar to the highly porous and catalytically active Pd films that can be generated very easily inside glass capillaries by the decomposition of Pd(OAc)_2_ under somewhat similar elevated temperature conditions [62]. Work by Organ and coworkers has demonstrated, that these Pd-on-glass films (and other metal-coated glass capillaries) can be used for a variety of synthetically important flow chemistry applications in an elevated temperature regime [63–64], including Mizoroki–Heck chemistry [62]. It should be emphasized that our interest in this steel-based immobilized catalytic Pd system was mainly to demonstrate reactor contamination/fouling and not of a preparative nature. After all, only 1.5 mg of Pd(OAc)_2_ were used to load a 20 m long stainless steel coil, a procedure clearly not suitable to sustain catalytic activity of the “palladated” reactor coil for an extended time [65–67].

Since the “Pd-on-steel” catalyst cannot be easily removed by washing with pure solvent and steel coils for most flow instruments are designed for multiple usage, a cleansing procedure was developed. After considerable experimentation, we found that the use of a KCN/*m*-nitrobenzenesulfinic acid sodium salt mixture in water [68] at 80 °C effectively removes all Pd from the surface of the steel coils as these coils were not catalytically active in subsequent Mizoroki–Heck chemistry. Only renewed loading of the coil by running a Mizoroki–Heck reaction with Pd(OAc)_2_ or by simply processing a Pd(OAc)_2_ solution through the coil at elevated temperature regenerated the “palladated” stainless steel coils.

### Influence of pressure on reaction rate in flow reactors

**Scheme 5:**
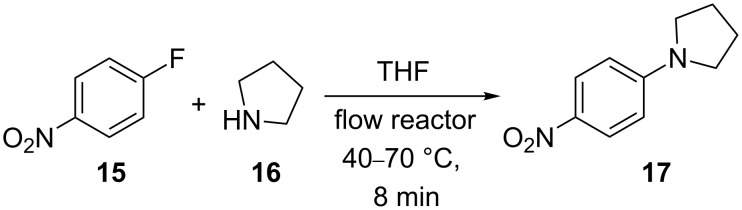
Nucleophilic aromatic substitution of 4-fluoro-1-nitrobenzene (**15**) with pyrrolidine (**16**) under continuous flow conditions.

**Figure 5:**
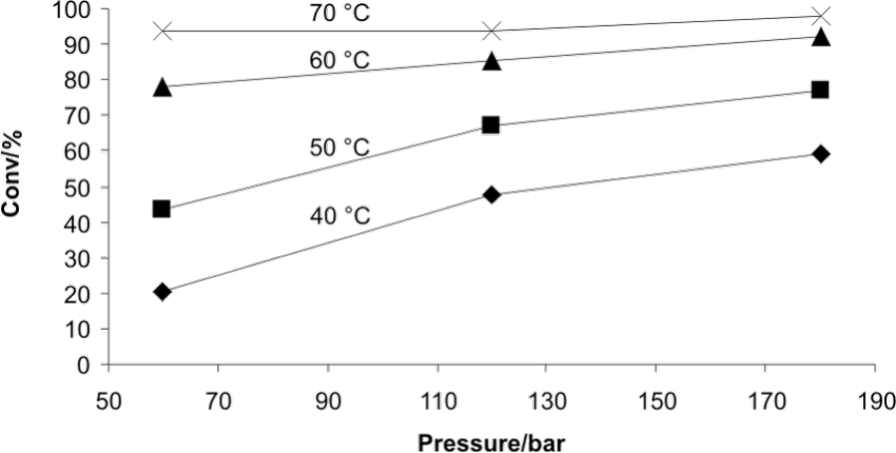
Nucleophilic aromatic substitution reaction of 1-fluoro-4-nitrobenzene (**15**) with pyrrolidine (**16**) in THF (Scheme 5) at different temperatures and three different pressures performed in a 8 mL stainless steel coil at a flow rate of 1 mL/min.

In the resistively heated flow reactor used in this work, the pressure during a flow experiment can be set in a range of 50–180 bar with the help of a back-pressure valve [24]. This allows the influence of pressure on the rate of chemical transformations under continuous flow conditions to be studied. While in the tetrazole decomposition described earlier, reaction pressure apparently had no significant influence, there are some transformations that can potentially be influenced by reaction pressure under these flow conditions. In general, the rate and equilibrium of many chemical reactions can be influenced when pressures in the range of 1–20 kbar are applied. Typically, reactions that are accompanied by a decrease in molar volume can be accelerated by increasing pressure (Δ*V*^≠^ < 0) and the equilibria are shifted towards the side of the products (reaction volume Δ*V* < 0) [69–70]. A variety of high-pressure flow chemistry examples have been reported in the literature, but the number of cases where a pressure influence is seen in the medium pressure range (50–200 bar) accessible in standard flow reactors is rather limited [69–70]. One transformation where pressure-dependent rate enhancements (1–600 bar) have been observed in a 3 μL fused-silica capillary, albeit not under continuous flow conditions, is the nucleophilic aromatic substitution reaction of 4-fluoro-1-nitrobenzene (**15**) with pyrrolidine (**16**) in THF (Δ*V*^≠^ = −58 cm^3^/mol) (Scheme 5) [71]. In order to confirm that these pressure enhancements can also be experienced in a stainless steel mesofluidic flow reactor, the nucleophilic substitution was repeated in an X-Cube Flash instrument. Employing an 8 mL steel coil and an overall flow rate of 1 mL/min (two individual reagent streams at 0.5 mL/min each, residence time = 8 min), the conversion of the substitution reaction at 40 °C was determined at 60, 120 and 180 bar pressure. Indeed, for this transformation a higher reaction pressure increases the reaction rate as previously observed in a fused-silica capillary (Figure 5) [71]. Since the mesofluidic reactor not only allows variation of pressure but also temperature, a series of experiments was designed in which temperature and pressure were increased at the same time. As the data presented in Figure 5 demonstrate, a significant influence of reaction pressure on reaction rate is no longer observable at a temperature of 70 °C.

## Conclusion

In summary, we have demonstrated that the high-temperature decomposition of 5-benzhydryl-1*H*-tetrazole (**2**) in a NMP/AcOH/H_2_O 5:3:2 mixture is remarkably accelerated when performed in a resistively heated stainless steel coil as compared to either microwave batch experiments using Pyrex vessels or flow experiments where stainless steel or Hastelloy coils are heated by conductive techniques. Through a series of control experiments, effects of homogeneous metal catalysis as a result of reactor corrosion, and heterogeneous catalytic effects derived from the large metal surface area in the microreactor environment (wall effects) were excluded from being responsible for these unusual phenomena. Although no obvious explanation for these dramatic enhancements in reaction rates can be offered at this time, the results presented herein clearly demonstrate that the conversion of batch to flow chemistry can sometimes be a non-trivial affair and great care must therefore be taken in the interpretation of results.

In this context, we have also presented results of microreactor fouling studies that highlight the fact that reactor contamination, for example, by deposition of catalytically active transition metals inside a microreactor, must also be carefully considered in executing flow chemistry. In the case of a Mizoroki–Heck coupling, we have demonstrated that small amounts of Pd metal retained inside a stainless steel reactor can exhibit catalytic activity in subsequent chemical transformations. As most microreactors for high-temperature applications are made out of non-transparent materials, reactor contamination may not be immediately obvious.

Finally, the fact that stainless steel or Hastelloy-based flow reactors are sensitive to acids, even in small quantities, is sometimes disregarded. In particular at high temperatures, significant corrosion of the reactor material can result, leading to unwanted side reactions. In addition, in specific cases reaction pressure can influence reaction rates as evidenced by the nucleophilic aromatic substitution reaction of 1-fluoro-4-nitrobenzene with pyrrolidine in the 60–180 bar pressure range.

## Experimental

**General remarks: **^1^H NMR spectra were recorded on a Bruker 300 MHz instrument. Melting points were determined on a Stuart™ SMP3 melting point apparatus. Low resolution mass spectra were obtained on an Agilent 1100 LC/MS instrument using atmospheric pressure chemical ionization (APCI) in positive or negative mode. Analytical HPLC analysis was carried out on a Shimadzu LC-20 system with a LiChrospher 100 C18 reversed–phase analytical column (119 × 3 mm, 5 µm particle size) at 25 °C, using mobile phase A (water/MeCN 9:1 (v/v) + 0.1% TFA) and phase B (MeCN + 0.1% TFA), with linear gradient from 30% B to 100% B in 8 min and 2 min with 100% phase B. GC-FID analysis was performed on a Trace-GC (ThermoFisher) with a flame ionization detector with an HP5 column (30 m × 0.250 mm × 0.025 μm). After 1 min at 50 °C the temperature was increased in 25 °C/min steps up to 300 °C and kept at 300 °C for 4 min. The detector gas for the flame ionization was H_2_ and compressed air (5.0 quality). GC–MS conditions were as follows: injection temperature 250 °C, HP-5 MS column (30 m × 0.250 mm ID, 0.25 μm film); carrier gas helium 5.0, flow 1 mL min^−1^, temperature gradient programmed from 60 to 300 °C at 20 °C min^−1^ after an initial time of 6 min.

The MS conditions were as follows: positive EI ionization, ionization energy 70 eV, ionization source temperature 280 °C, emission current 100 μA. Flash chromatography separations were performed on a Biotage SP1 instrument with petroleum ether/ethyl acetate mixtures as eluent. Microwave irradiation experiments were carried out in an Anton Paar Monowave 300 instrument with appropriate internal fiber-optic temperature control [50–52]. All products synthesized in this study are known in the literature and have been characterized by ^1^H NMR and MS analysis. All solvents and chemicals were obtained from standard commercial vendors and were used without any further purification.

Pd concentrations from the leaching experiments of a “palladated” steel coil and the metal screening of the “NMP/AcOH/H_2_O mixtures” were determined after microwave assisted acid digestion in an MLS UltraClave III. The temperature was ramped up in 30 min to 250 °C and kept at this temperature for a further 30 min. Pd was quantitatively determined at *m*/*z* 105 with an Agilent 7500ce inductively coupled plasma mass spectrometer. A calibration was performed with an external calibration curve established from 1.000 g Pd/L standard (CPI International). Indium was used as the internal standard. The element screening in the digests of the “NMP/AcOH/H_2_O mixtures” was performed in the semi-quantitative analysis mode using the Merck VI ICPMS standard for calibration. For both measurements, the samples were dispatched from the autosampler via an integrated sample introduction system (ISIS) from Agilent Technologies to an Ari Mist HP nebulizer (Burgener Research International) and further into the ICPMS.

### 

#### Continuous flow experiments

The flow experiments described herein were performed in a Thales X-Cube Flash reactor (electric resistance heating) [24], a Uniqsis FlowSyn instrument (conductive heating) [25] or in a self-made flow device constructed by immersing the corresponding coil material – connected to a HPLC pump and back-pressure regulator – into a pre-heated oil bath. The flow reactors were equipped with coils made from different materials (SX 316L steel, Hastelloy C-4 or PTFE), of variable lengths and inner diameters. A system pressure of 140 bar was applied for all experiments in the X-Cube Flash reactor while a pressure of ≈34 bar was selected for all experiments in the FlowSyn instrument and in the self-made device. Experiments in PTFE coils were limited to system pressures of ≤ 14bar.

#### Tetrazole decomposition in flow

The reactor was heated to the selected temperature while the solvent mixture (e.g., NMP/AcOH/H_2_O 5:3:2) was pumped through the reactor at the desired flow rate. After the temperature was stable, the feed was switched from pure solvent to reagent (e.g., 0*.*125 M solution of tetrazole **2** in NMP/AcOH/H_2_O 5:3:2). A defined volume (≈1 mL) of the reagent solution was introduced into the reactor and the feed was then changed back to pure solvent. The outcoming processed reagent solution was collected. For the kinetic analysis of the decomposition process (Figure 4 and Figure S5, Supporting Information File 1), the flow rate was varied and, after equilibration, the feed was switched back from pure solvent to reagent. The processed mixture was collected and the procedure repeated until enough data points were collected. For reaction monitoring and kinetic analysis, 100 µL samples were taken from the collected mixtures, diluted with 0.9 mL MeCN and analyzed by HPLC-UV at 215 nm. A detailed description of the isolation and characterization of decomposition products (Scheme 2) is given in the Supporting Information File 1.

#### Tetrazole synthesis in flow (Scheme 1)

The first attempts to synthesize tetrazole **2** (Figure 1) were performed in a resistively heated 8 mL coil at 220 °C with a residence time of 16 min (0.5 mL/min flow rate). The reagent mixture was a 0.67 M solution of diphenylacetonitrile (**1**) with 2.5 equiv of NaN_3_ in NMP/AcOH/H_2_O 5:3:2.

#### Tetrazole decomposition using microwave conditions

A 2.5 mL sample of a 0.125 M solution of tetrazole **2** in NMP/AcOH/H_2_O (5:3:2) in a 10 mL microwave process vial with a stirrer bar was heated in the microwave reactor (Monowave 300) at the indicated temperature (Figure 3 and Figures S1–S4, Supporting Information File 1). For reaction monitoring the vial was cooled to 60 °C. After a defined time, 40 μL samples were taken with a transfer pipette, the sample diluted with 1.0 mL of MeCN and analyzed by HPLC at 215 nm. The experiments were carried out either in a standard 10 mL Pyrex tube or a vessel made from sintered silicon carbide and gave identical results.

#### Bechamp reduction (Table 1)

A 0 M, 0.25 M, 0.5 M and 1 M aqueous HCl solution in ethanol was prepared from conc. HCl and ethanol. A sample of 1 mmol (123.1 mg) of nitrobenzene (**10**) was added in a graduated cylinder and the volume was made up to 2 mL with the respective HCl solution to obtain a 0.5 M solution of nitrobenzene. The flow experiments were performed at 150 °C in coils made from stainless steel, Hastelloy or PTFE. The flow rates for the different coils were selected in order that the residence time was 20 min in every coil. The feed was switched between pure ethanol and reagent solutions.

#### Heck–Mizoroki coupling using palladated steel coils. Loading procedure

4-Iodobenzonitrile (150 mg, 0.65 mmol), *n*-butyl acrylate (126 mg, 142 mL, 0.98 mmol, 1.5 equiv), Pd(OAc)_2_ (1.5 mg, 0.0065 mmol, 1 mol %) and triethylamine (100 mg, 137 mL, 0.98 mmol, 1.5 equiv) were mixed together with MeCN (2 mL) into a 5 mL glass vial and stirred for 2 min. The X-Cube Flash instrument was equipped with a stainless steel reaction coil (16 mL volume, 10 min residence time at 1.6 mL/min flow rate). The reaction parameters – temperature (180 °C), flow rate (1.6 mL/min), and pressure (55 bar) – were selected on the flow reactor and processing was started, whereby only pure solvent was pumped through the system until the instrument had achieved the desired reaction parameters and stable processing was assured. At that point, the inlet tube was switched to the vial containing the freshly prepared reaction mixture. After processing through the flow reactor, the inlet tubing was dipped back into a vial with pure solvent and processed for further 10 min, thus washing the system from any remaining reaction mixture. The processed reaction mixture was then combined with the washings and the solvent was removed under vacuum. The residue was dissolved in acetone (2 mL) and transferred to a silica-samplet, dried for 2 h at 70 °C in a drying oven, and then subjected to automated flash chromatography with petroleum ether/ethyl acetate (0 to 45% gradient) as eluent to provide 141 mg (94%) of cinnamic ester **14**, identical in all respects with a previously prepared sample in our laboratories [20].

#### Pd leaching studies

An identical experiment was performed in the loading procedure, but in the absence of the Pd catalyst employing the “palladated” coil described earlier. After complete processing through the flow reactor, the inlet tubing was dipped back into a vial with pure solvent and processed for further 10 min, thus washing any remaining reaction mixture from the system. A 10 mL sample was collected and the solvent removed under vacuum before submission for ICPMS analysis. Conversion was determined by the means of GC-MS. In a similar manner 10 mL fractions of solvent were collected at room temperature and at 180 °C, the solvent evaporated and the residues submitted to ICPMS analysis.

#### Pd cleaning procedure

The X-Cube Flash instrument was equipped with a “palladated” stainless steel reaction coil (16 mL volume, 10 min residence time at 1.6 mL/min flow rate). The reaction parameters – temperature (80 °C), flow rate (1.6 mL/min), and pressure (55 bar) – were selected on the flow reactor and processing was started, whereby only distilled water was pumped through the system until the instrument had achieved the desired reaction parameters and stable processing was assured. At that point, the inlet tube was switched to the vial containing 100 mL freshly prepared mixture of KCN (10 g/L) and *m*-nitrobenzenesulfinic acid sodium salt (10 g/L) in water. After processing of the 100 mL through the flow reactor (ca. 60 min), the inlet tubing was dipped back into a vial with distilled water and processed for a further 10 min. The process was repeated two more times with fresh 100 mL portions of KCN/*m*-nitrobenzenesulfinic acid sodium salt in water mixture before the reactor was finally washed with distilled water for 30 min to remove any remaining residues of the KCN/*m*-nitrobenzenesulfinic acid sodium salt in water mixture.

#### Pressure dependence of nucleophilic aromatic substitution (Scheme 5)

The reaction parameters – temperature (40 °C), flow rate (0.5 mL/min), and pressure (60 bar) – were selected on the X-Cube Flash flow reactor, equipped with an 8 mL stainless steel reaction coil and processing was started with pure THF. After the instrument had achieved the desired reaction parameters and stable processing was assured, freshly prepared solutions of 1-fluoro-4-nitrobenzene (**15**) (904 mg, 680 µL, 6.41 mmol) in 25 mL of THF and pyrrolidine (**16**) (4.56 g, 5.35 mL, 64.1 mmol) in 25 mL THF were introduced separately into the coil at flow rates of 0.5 mL min^−1^ utilizing two pumps to give a total flow rate of 1 mL/min (8 min residence time). After processing 5 mL of the reaction mixture through the flow reactor, the inlet tubings were dipped back into the vials with pure solvent and a sample was collected after an appropriate time. To a 100 µL portion of this collected sample, 500 µL of 2 M HCl were immediately added to quench the reaction. Then 500 µL of water and 600 µL DCM were added to extract the product and unreacted nitrobenzene**.** After vigorous shaking and settling, 100 µL of the organic layer was then diluted with 1 mL of MeCN before injecting into the HPLC for an offline analysis. The pressure was increased to 120 bar and then to 180 bar to collect two more samples at 40 °C. Similarly, the above steps were repeated to collected data for temperatures of 50, 60 and 70 °C (Figure 5).

## Supporting Information

File 1 Details of experimental procedures, kinetic analysis and spectral data.
