# Supplementary material for: Unusual behavior in the reactivity of 5-substituted-1H-tetrazoles in a resistively heated microreactor
Source: Beilstein J Org Chem. 2011 Apr 21;7:503–17. doi: 10.3762/bjoc.7.59 (PMC3107459; doi:10.3762/bjoc.7.59)
Supplement: File 1 — Details of experimental procedures, kinetic analysis and spectral data. [file Beilstein_J_Org_Chem-07-503-s001.pdf]

Supporting Information

for

Unusual behavior in the reactivity of 5-substituted-1H-tetrazoles in a resistance-heated microreactor

Bernhard Gutmann[1], Toma N. Glasnov[1], Tahseen Razzaq[1], Walter Goessler[2], Dominique M. Roberge[3] and C. Oliver Kappe*[1]

Address: [1]Christian Doppler Laboratory for Microwave Chemistry (CDLMC) and Institute of Chemistry, Karl-Franzens-University Graz, Heinrichstrasse 28, A-8010 Graz, Austria and [2]Institute of Chemistry, Analytical Chemistry, Karl-Franzens-University Graz, Universitätsplatz 1, A-8010 Graz, Austria and [3]Continuous Flow/Microreactor Technologies, Lonza AG, CH-3930 Visp, Switzerland

Email: C. Oliver Kappe* - oliver.kappe@uni-graz.at

* Corresponding author

Details of experimental procedures, kinetic analysis and spectral data

Decomposition of tetrazole 2. Synthesis and characterization of intermediates

*N'-Acetyl-2,2-diphenylacetohydrazide (**7**):* A sample of diphenylacetic acid (212 mg, 1.0 mmol) (**8**) was dissolved in 2 mL of 0.5 M HCl in MeOH in a 5 mL microwave process vial equipped with a magnetic stir bar. The vial was sealed by capping with a Teflon septum fitted in an aluminum crimp top and irradiated in a single-mode microwave reactor at 150 °C (~15 bar) until diphenylacetic acid was fully converted to its methyl ester (30 min). The solvent was removed under reduced pressure, 3 mL of hydrazine hydrate were added, the vial was re-sealed and re-heated in the microwave reactor at 150 °C (~12 bar) until the ester was consumed (65 min). The reaction mixture was extracted repeatedly with 10 mL of saturated $NaHCO_3$ and 10 mL ethyl acetate. The organic phases were collected, dried over $MgSO_4$ and the solvent was removed under reduced pressure to afford 147 mg (67%) of pure 2,2-diphenylacetohydrazide; ^{1}H NMR (300 MHz, $CHCl_3$, TMS) δ = 3.97 (s, 2H, NH_2), 4.91 (s, 1H, CH), 6.91 (br s, 1H, C(O)NH) 7.27–7.38 (m, 10H, aromatic). A sample of the 2,2-diphenylacetohydrazide (113 mg, 0.5 mmol) was dissolved in 2 mL of $CDCl_3$ in a round bottomed flask and 57 µL (0.60 mmol, 1.2 equiv) acetic anhydride was added. The flask was loosely closed and the reaction mixture stirred at room temperature for 24 h. The reaction mixture was extracted repeatedly with 10 mL of saturated $NaHCO_3$ and 10 mL EtOAc. The organic phases were collected, dried over $MgSO_4$, and the solvent was removed under reduced pressure to afford 113 mg (84%) of *N'*-acetyl-2,2-diphenylacetohydrazide (**7**) as a white solid: Alternatively, *N'*-acetyl-2,2-diphenylacetohydrazide was synthesized by heating 59 mg of 2-benzhydryl-5-methyl-1,3,4-oxadiazole (**6**) in 1 mL AcOH/H_2O in a microwave reactor for 15 min at 180 °C (10 bar). Mp 217–218 °C, ^{1}H NMR (300 MHz, DMSO-d_6, TMS) δ = 1.85 (s, 3H, CH_3), 4.99 (s, 1H, CH), 7.23–7.33 (m, 10H, aromatic), 9.88 (s, 1H, C(O)NH), 10.28 (s, 1H, C(O)NH); MS (neg-ESI): *m*/*z* (%): 267 (100) $[M + H]^-$.

*2,2-Diphenylacetic acid (**8**):* The NMP/AcOH/H_2O mixtures resulting after flow or microwave processing were extracted repeatedly with 10 mL of saturated $NaHCO_3$ and 10 mL of ethyl acetate. The pH of the aqueous solution was adjusted to ~pH 1 with conc. HCl. The mixture was cooled in an ice-bath, the precipitate collected by filtration and washed thoroughly with cold 1N HCl to furnish diphenylacetic acid (**8**) as white needles. The isolated compound was identical in all respects to a commercial sample. ^{1}H NMR (300 MHz, DMSO-d_6, TMS) δ = 5.07 (s, 1H, CH), 7.31–7.33 (m, 10H, aromatic), 12.60–12.90 (br s, 1H, COOH).

*Diphenylmethane (**3**):* The NMP/AcOH/H_2O mixtures (~4 mL) resulting from extended tetrazole decomposition in microwave or flow format containing essentially pure diphenylmethane (HPLC-UV) were extracted repeatedly with 10 mL of saturated $NaHCO_3$ and 10 mL $CHCl_3$. The

organic phases were collected, dried over $MgSO_4$, and the solvent was removed under reduced pressure to afford 61 mg (73%) of diphenylmethane as a clear liquid. The isolated compound was identical in all respects to a commercial sample. ^{1}H NMR (300 MHz, $CDCl_3$, TMS) δ = 4.02 (s, 2H, CH_2), 7.21–7.35 (m, 10H, aromatic).

*2-Benzhydryl-5-methyl-1,3,4-oxadiazole (**6**):* A sample of tetrazole **2** (118 mg, 0.50 mmol) was dissolved in 5 mL of Ac_2O and heated under reflux (~140 °C) in a round bottomed flask equipped with a reflux condenser and a $CaCl_2$ drying tube until the tetrazole was fully consumed (~16 h). The Ac_2O solvent was removed under reduced pressure to afford crude oxadiazole **6** in quantitative yield as a brownish solid. Mp 114–115 °C, lit. [1] mp 115.5–117 °C; ^{1}H NMR (300 MHz, DMSO-d_6, TMS) δ = 2.52 (s, 3H, CH_3), 5.71 (s, 1H, CH) 7.29–7.36 (m, 10H, aromatic); MS (pos-ESI): *m*/*z* (%): 251 (100) $[M + H]^-$.

*2,2-Diphenylacetamide (**9**):* A sample of diphenylacetonitrile (**1**) (97 mg, 0.50 mol) was dissolved in 2 mL of conc. H_2SO_4 and stirred at room temperature until the nitrile was completely consumed (~24 h). The reaction mixture was extracted repeatedly with 10 mL of saturated $NaHCO_3$ and 10 mL EtOAc. The organic phases were collected, dried over $MgSO_4$, and the solvent was removed under reduced pressure to afford 2,2-diphenylacetamide (**9**) as a white solid. mp 165–166 °C, lit. [2] mp 169 °C; ^{1}H NMR (300 MHz, $CDCl_3$, TMS) δ = 4.98 (s, 1H, CH), 5.51–5.66 (br s, 1H, C(O)NH), 5.81–5.96 (br s, 1H, C(O)NH), 7.29–7.36 (m, 10H, aromatic); MS (pos-ESI): *m*/*z* (%): 212 (100) $[M + H]^-$.

Table S1: ICPMS analysis in µg L^{-} of the NMP/AcOH/H_2O mixtures flowed through different metal coils at 220 °C for 5 min.[a]

[µg L^{-1}]	V	Cr	Mn	Fe	Co	Ni	Cu	Zn	Nb	Mo	Cd	Sn	Ce	W
Blank[b]	3±1	14±2	4±2	1730 ±60	< 5	18±5	15 ±1	210 ±60	< 5	11±5	< 5	< 5	< 5	8±4
Hastelloy[c]	5.1 ±0.1	1600 ±360	45±8	2170 ±150	20±4	5300 ±1100	14 ±2	190 ±15	40 ±8	2000 ±400	< 5	< 5	69 ±14	2370 ±550
SX 316L[d] 20 ml coil	49 ±2	13000 ±0	74±6	5400 ±170	14±1	1330 ±60	24 ±10	330 ±100	< 5	3700 ±200	6±1	12 ±1	32 ±2	36±2
SX 316L[e] 4 ml coil (1)	50 ±10	12700± 3100	1270 ±310	33300 ±7100	99 ±19	9100 ±1760	33 ±4	220 ±80	< 5	740 ±170	< 5	< 5	< 5	4.1 ±0.4
SX 316L[e] 4 ml coil (2)	113 ±6	32300± 1500	3000 ±100	80700 ±4000	240 ±10	21000 ±1000	12 ±1	210 ±110	< 5	1430 ±60	< 5	< 5	< 5	9±1
SX 316L[e] 8 ml coil	8,0 ±0.5	1030 ±60	4900 ±300	113300 ±5800	190 ±10	15700 ±580	19 ±1	330 ±100	< 5	92 ±7	< 5	8±1	140 ±10	2.0 ±0.4
SX 316L[e] 16 ml coil	70 ±20	19000 ±4600	1600 ±420	50700 ±12900	150 ±40	13700 ±3100	21 ±2	210 ±75	< 5	260 ±60	< 5	< 5	45 ±10	4±3

[a]Reaction conditions: The flow reactor was heated to the selected temperature while the NMP/AcOH/H_2O 5:3:2 solvent mixture was pumped through the reactor. The flow rates for the different coils were selected in order that the residence time was 5 min in every coil (flow rate in mL min^{-1} = reactor volume in mL divided by the residence time in min). After the temperature was stable, 20 mL of the out-coming processed solvent mixture was directed to "Waste". The following 20 mL processed solvent mixture was collected for ICPMS analysis.

[b]untreated NMP/AcOH/H_2O (5:3:2) mixture. [c]5.21 ml Hastelloy C-4 coil (i.d. 2 mm) heated in an oil-bath preheated to 220 °C. The coil was connected to a HPLC-pump and a 500 psi (~34 bar) back-pressure regulator.

[d]heated on a conductively heated Al block (Uniqsis FlowSyn). [e]heated by electrical resistance heating (Thales X-Cube Flash).

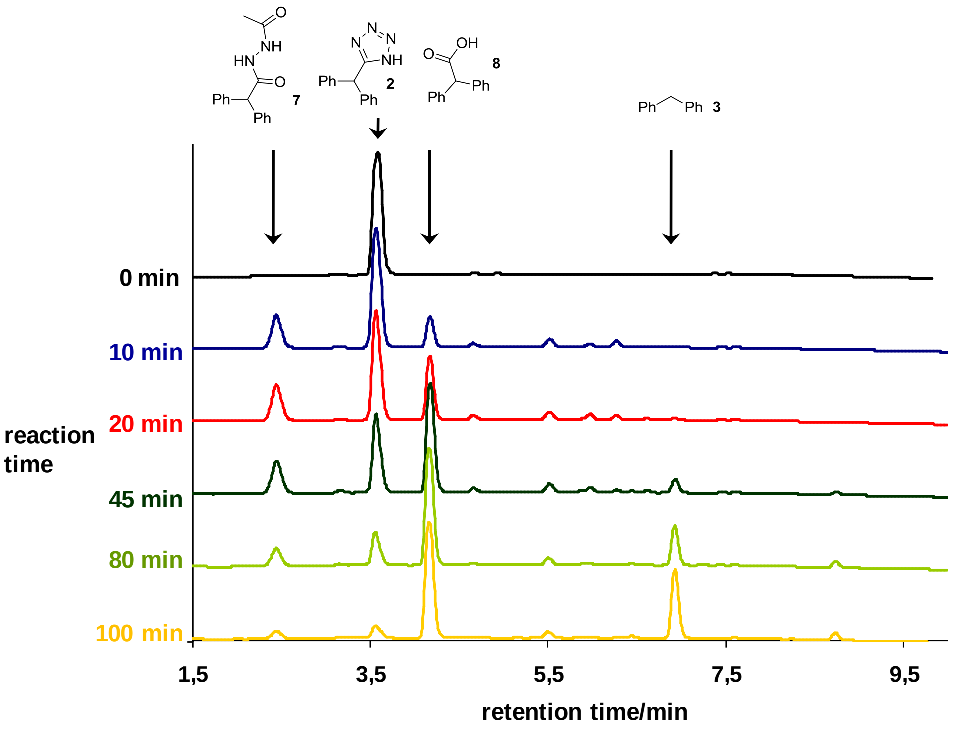

Figure S1: HPLC-UV chromatograms (215 nm) showing the decomposition of tetrazole **2** in NMP/AcOH/H_2O 5:3:2 (0.125 M) at different reaction times at 240 °C under microwave conditions.

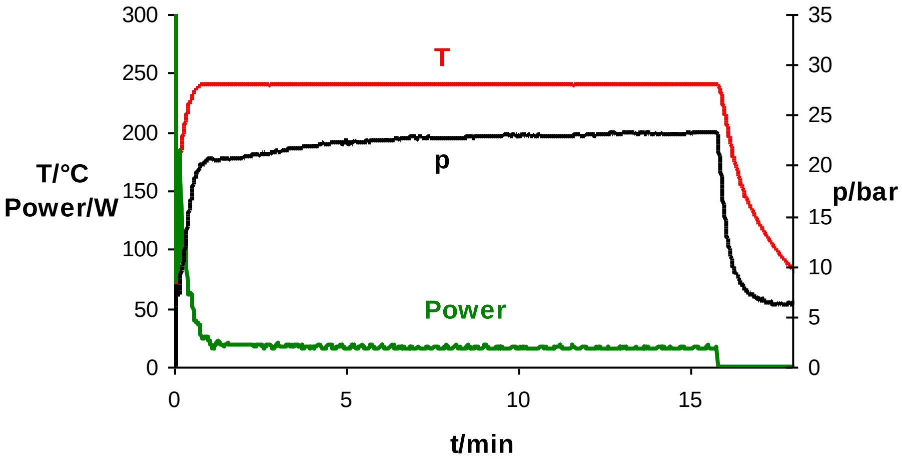

Figure S2: Internal fiber-optic temperature (T), pressure (p) and microwave power profile (Power) for the decomposition of tetrazole **2** at 240 °C using a batch microwave reactor (Monowave 300).

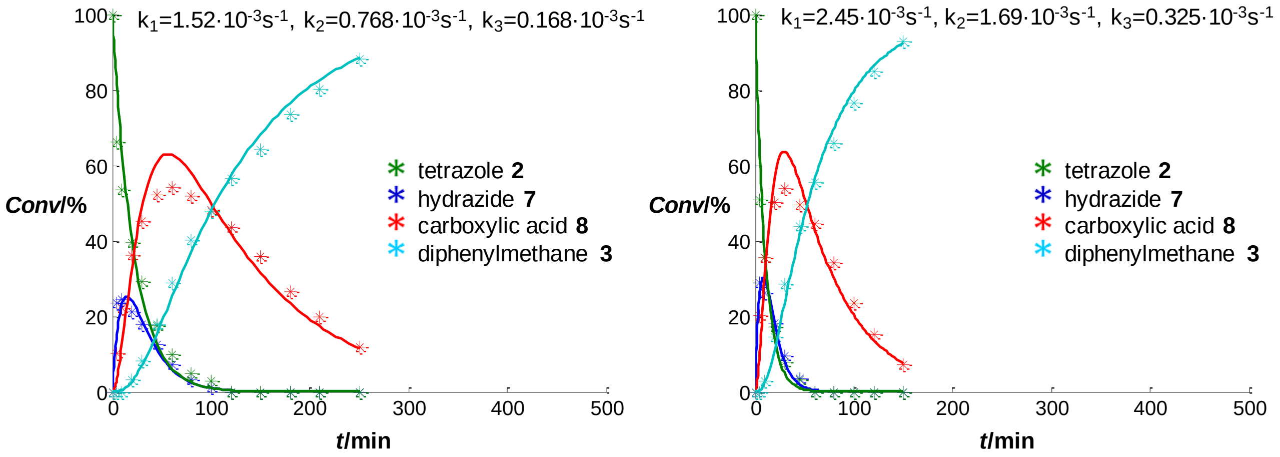

Figure S3: Decomposition of tetrazole **2** at 250 °C (left) and 260 °C (right) in a NMP/AcOH/H_2O 5:3:2 mixture (0.125 M); dots: experimental results; solid lines: predicted with a rate law according to Scheme 3. Conversions in percent were derived from HPLC-UV (215 nm) peak area integration without correction for response factors. The corresponding conversion for 240 °C is shown in Figure 3.

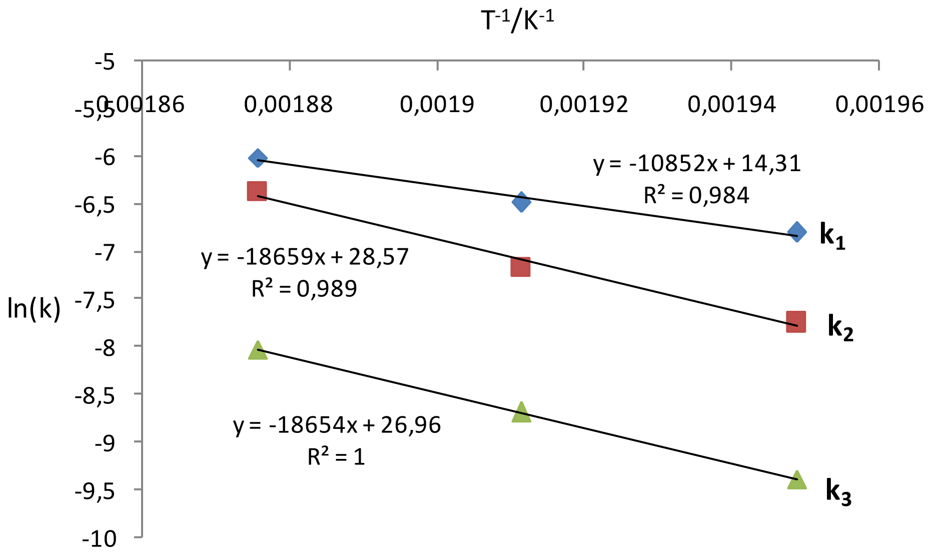

Figure S4: The ln(k) vs. T^{-1} plot for the rate constants from Figure 3 and Figure S3.

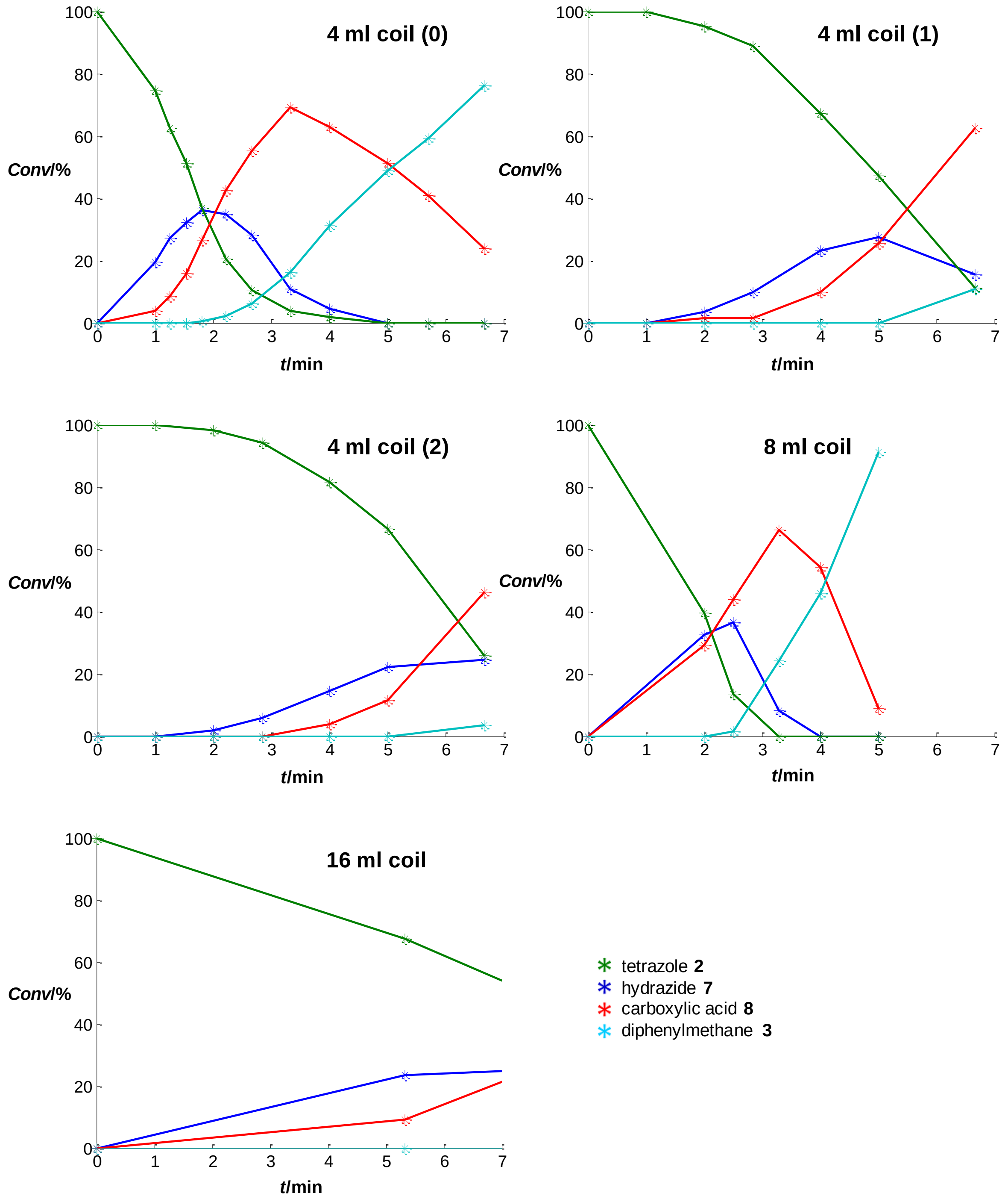

Figure S5: Decomposition of tetrazole **2** in 4 (3 different coils), 8 and 16 mL 1.0 mm i.d. resistance heated stainless steel coils at nominal 220 °C temperature in a NMP/AcOH/H_2O 5:3:2 mixture (0.125 M). Conversions in percent were derived from HPLC-UV (215 nm) peak area integration without correction for response factors. All coils have been used previously for a wide range of chemical transformations: The age/history of the coils apparently plays some role in the decomposition rate. In contrast to the microwave batch experiment, the decomposition steps are obviously not simple first order.

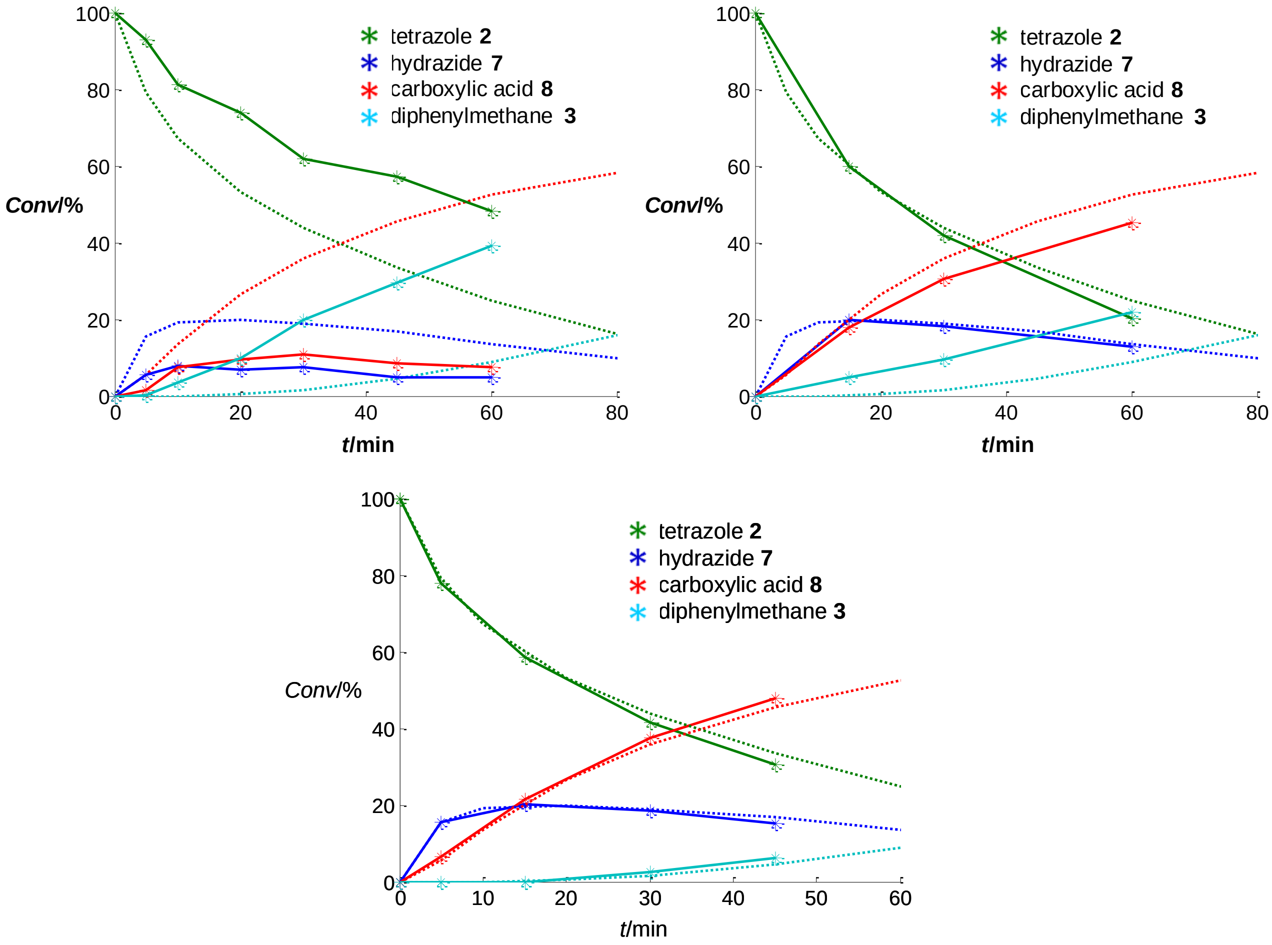

Figure S6: Decomposition of tetrazole **2** at 240 °C under batch microwave conditions in a NMP/AcOH/H_2O 5:3:2 mixture (0.125 M). **Top left:** solid lines: with stoichiometric amounts of Fe_2O_3; **Top right:** solid lines: with catalytic amounts of $Pd(OAc)_2$; **Bottom:** solid lines: decomposition in a silicon carbide microwave vial; dashed lines: decomposition in a glass vial without any additives for comparison. Conversions in percent were derived from HPLC-UV (215 nm) peak area integration without correction for response factors.

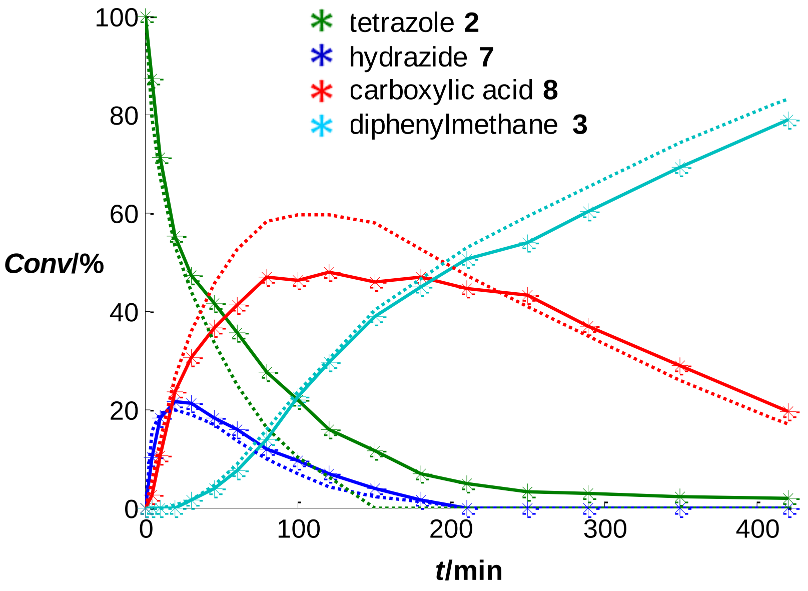

Figure S7: Decomposition of tetrazole **2** at 240 °C under batch microwave conditions in a NMP/AcOH/H_2O 5:3:2 mixture (0.125 M). Solid lines: the NMP/AcOH/H_2O mixture was previously processed through an 8 mL resistance heated stainless steel coil for 5 min at 220 °C before the decomposition experiment. Dashed lines: "fresh" NMP/AcOH/H_2O 5:3:2 mixture.

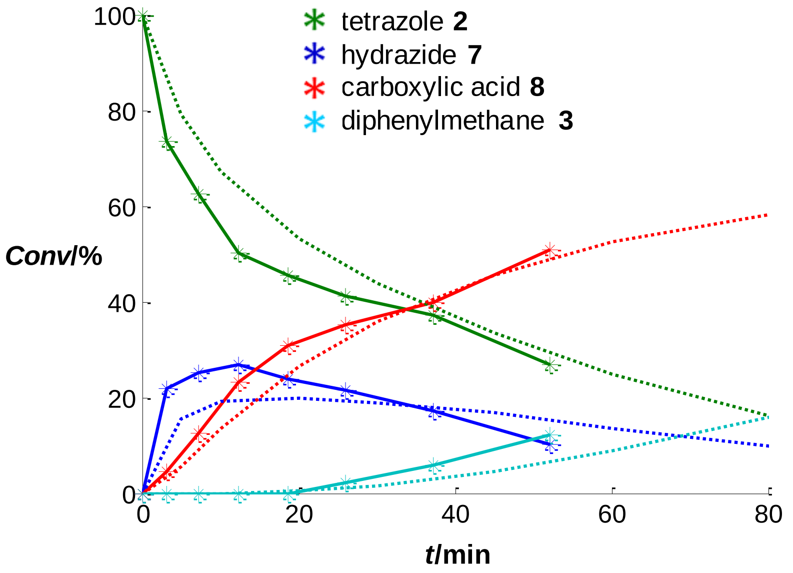

Figure S8: Comparison of tetrazole **2** decomposition at 240 °C under batch microwave and continuous flow Hastelloy conditions in a NMP/AcOH/H_2O 5:3:2 mixture (0.125 M). Solid lines: decomposition in a 5.21 mL Hastelloy coil; Dashed lines: decomposition under Pyrex batch microwave conditions.

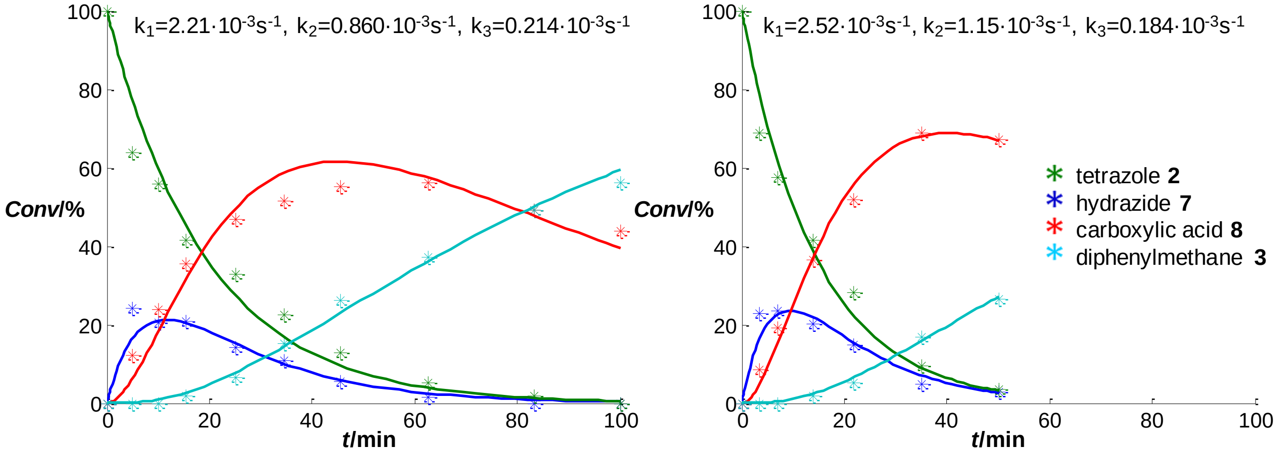

Figure S9: Decomposition of tetrazole **2** in two different conductively heated stainless steel coils (Uniqsis FlowSyn) at nominal 240 °C temperature in a NMP/AcOH/H_2O 5:3:2 mixture (0.125 M). Dots: experimental values; solid lines: predicted with the rate law according Scheme 3. The decomposition rates are nearly identical in both stainless steel coils but somewhat faster compared to the decomposition in the microwave reactor at the same temperature (Scheme 3).

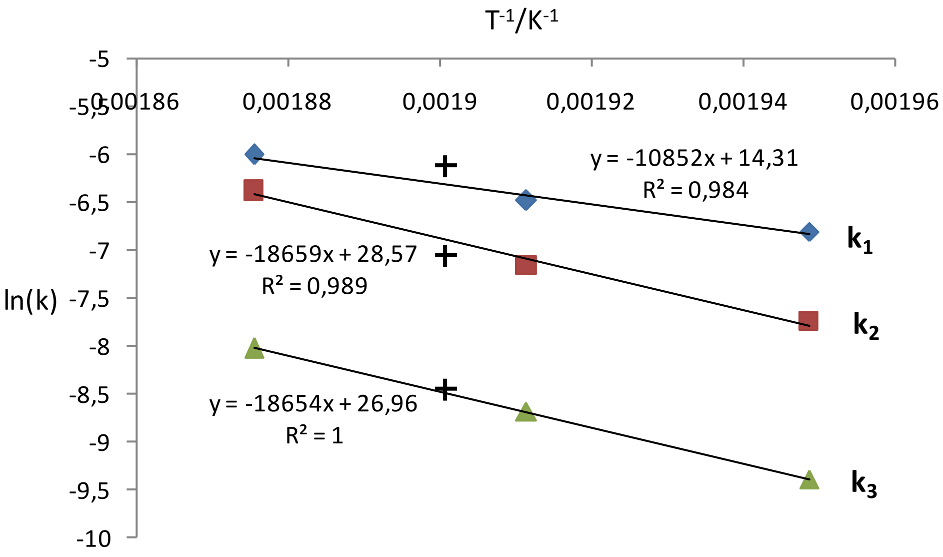

Figure S10: The rate constants obtained in the 20 mL stainless steel coil heated by conduction principles (Uniqsis FlowSyn) fit quite well in the ln(k) versus T^{-1} plot presented in Figure S4 assuming that the real temperature in these experiments was 253 °C (marked with black crosses). This strongly suggests that an imprecise temperature calibration, rather than metal or surface catalysis, is responsible for the observed small differences in the decomposition rates in the conductively heated flow reactor and the microwave batch reactor.

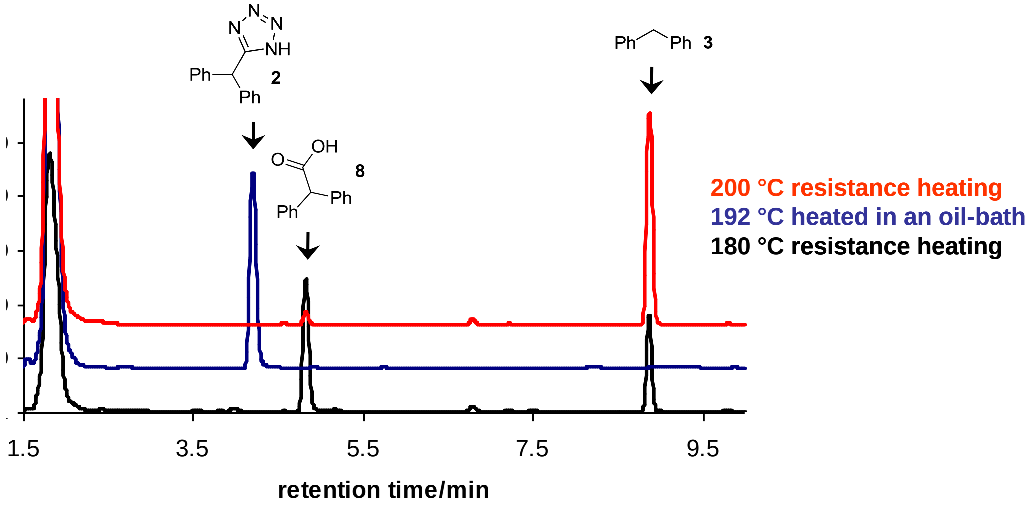

Figure S11: Comparison of tetrazole **2** decomposition in an 8 mL stainless steel coil at different temperatures in a NMP/AcOH/H_2O 5:3:2 mixture (0.125 M) after a residence time of 10 min. The same coil was used for all 3 experiments, however, the coil was heated either by resistance heating (180 °C and 200 °C) or the coil was heated in an oil-bath on a hot plate.

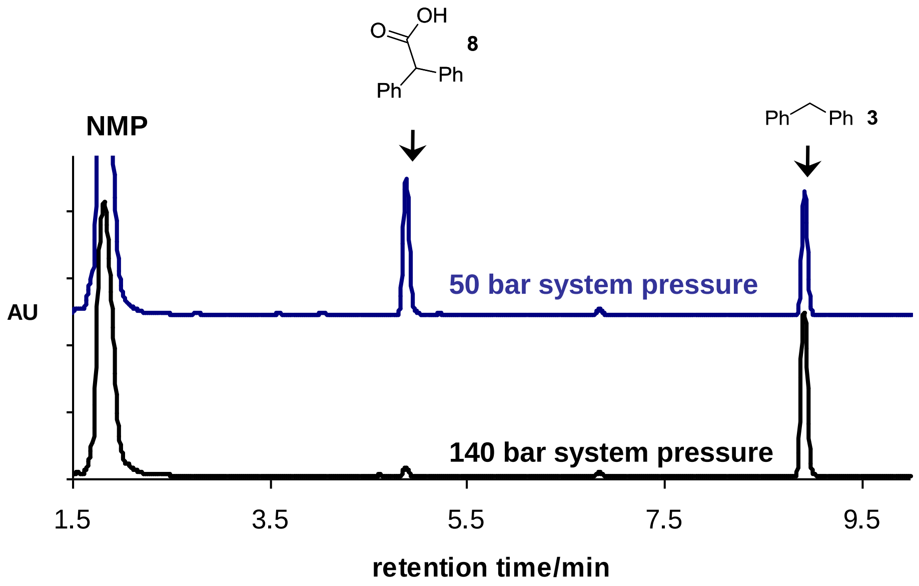

Figure S12: Effect of pressure on the decomposition experiments in an 8 mL stainless steel coil (Thales X-Cube Flash, 200 °C, 1 ml min^{-1} flow rate). The residence time of the reaction mixture for the experiment using 50 bar system pressure was ~ 7 min and hence about 1 min shorter as it should be. The substantial amounts of gases formed during the decomposition of the tetrazole propelled the reaction mixture out of the reactor (compared to the 140 bar experiment). The decomposition was therefore not complete because of the reduced residence time in the reactor, not because of a genuine pressure effect.

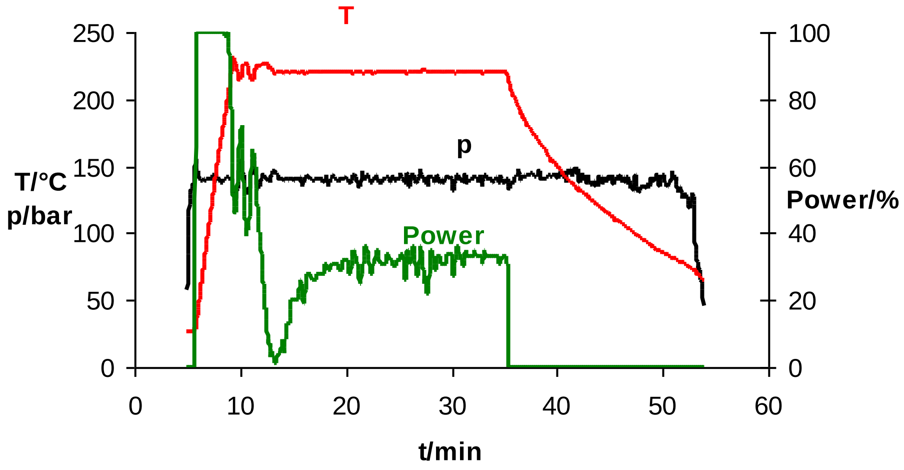

Figure S13: Reactor averaged temperature (T), system pressure (p) and generator power (Power) for decomposition experiments in an 8 mL stainless steel coil (Thales X-Cube Flash). No temperature or power fluctuations were noticed in these experiments.

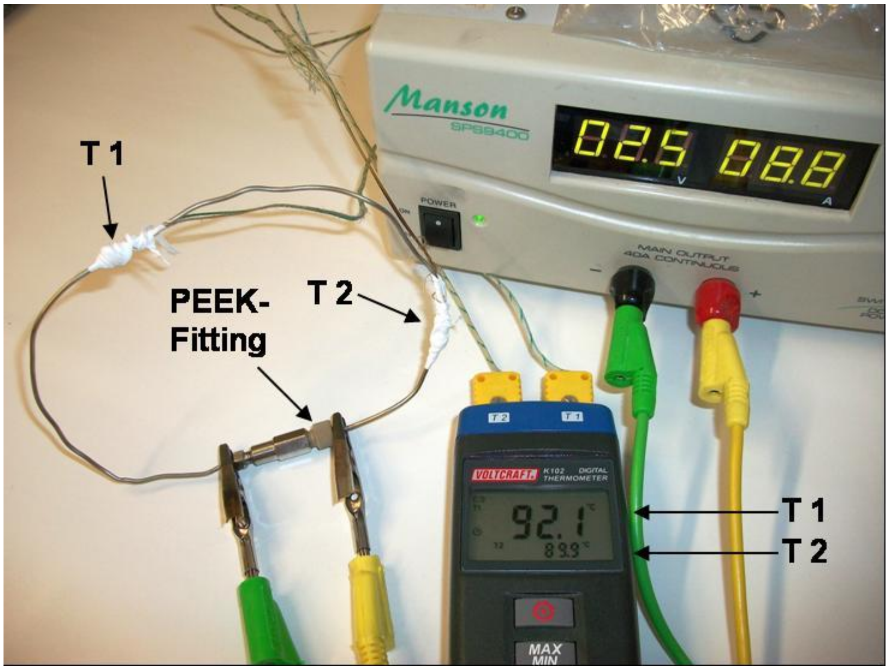

Figure S14: Experimental setup for the heating of a stainless steel capillary (~ 300 µL) by direct electrical resistance heating with a DC-switching power supply. One of the fittings is made of an insulator (PEEK) to prevent an electric short circuit between the two connectors. Two thermocouples measure the temperature on the outer surface of the stainless steel tubing at two different points.

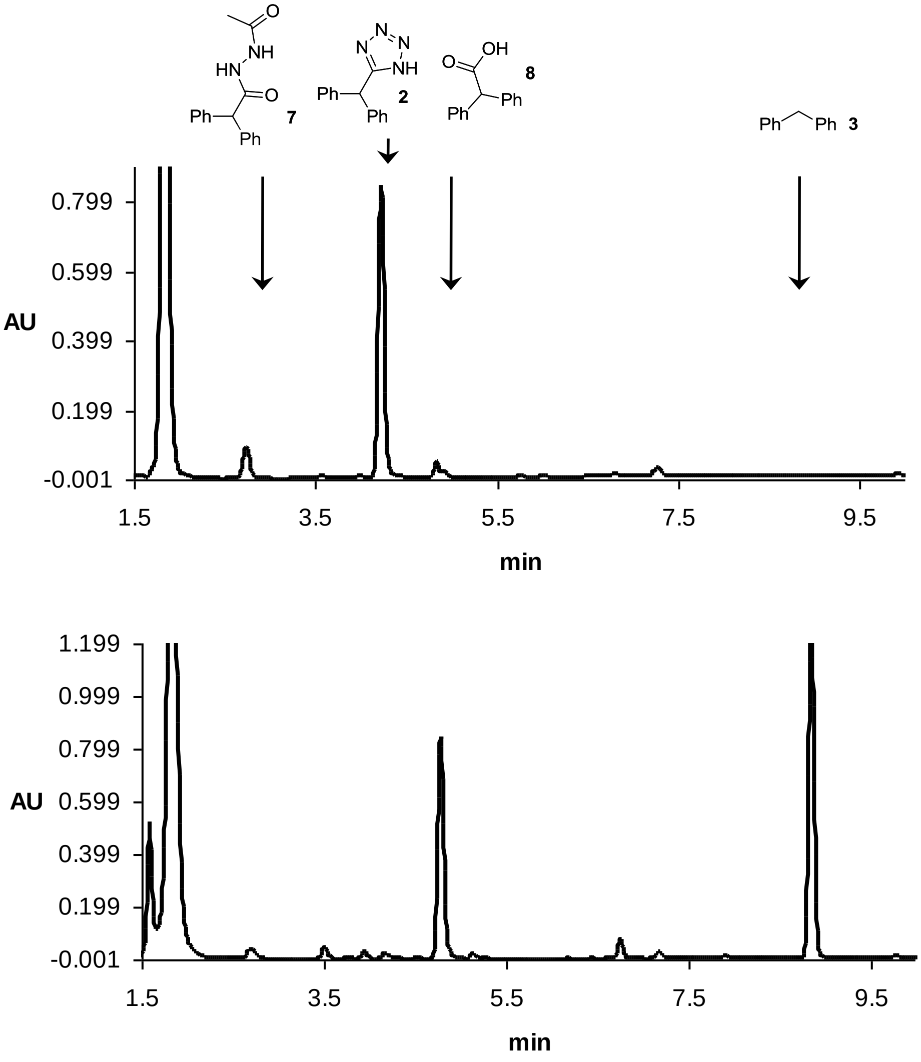

Figure S15: Decomposition of the tetrazole **2** in the 300 µL coil (0.125 M in a NMP/AcOH/H2O 5:3:2 mixture) after 10 min at 220 °C. The coil was heated either by direct electrical resistance heating (bottom) or in a conventional preheated oil-bath (top). The difference in decomposition behavior between the two heating modes at the same temperature is clearly evident.

HCl in mol/l	0.0 M	0.25 M	0.50 M	1.0 M

Stainless steel

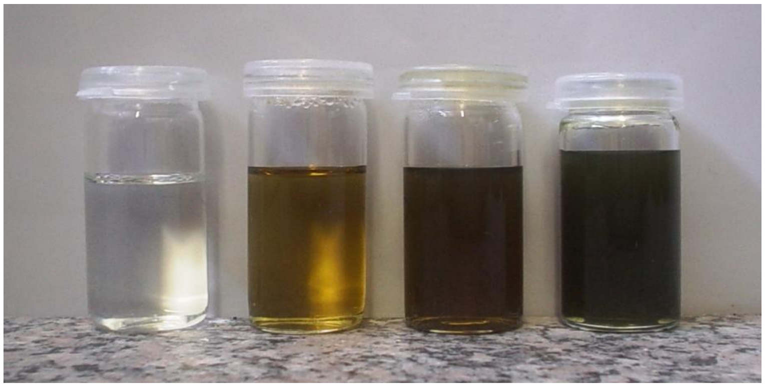

Aniline	0%	5%	14%	31%

HCl in mol/l	0.0 M	0.25 M	0.50 M	1.0 M

Hastelloy

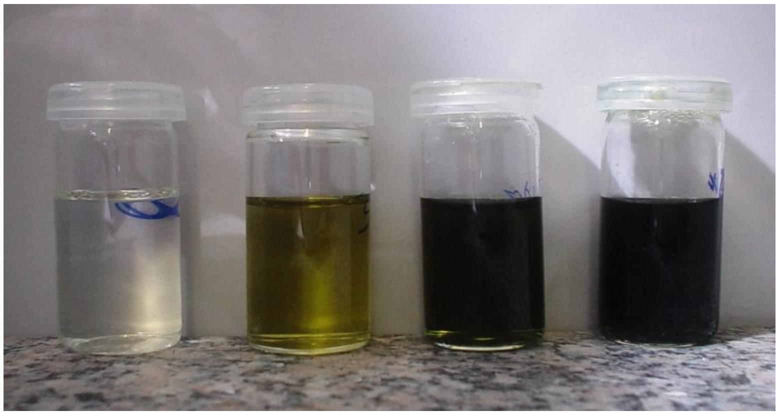

Aniline	0%	2%	7%	19%

Figure S16: Images of nitroreduction reaction mixtures (Table 1) obtained after flow processing through stainless steel and Hasteloy coils with varying concentrations of HCl. The green color is probably due to $Fe^{II/III}$ salts.

References

1. Huisgen, R. *Chem. Ber.* **1960,** *93,* 2106. doi:10.1002/cber.19600930932

2. Bellale, E. V.; Bhalerao, D. S.; Akamanchi, K. G. *J. Org. Chem.* **2008,** *73,* 9473–9475. doi:10.1021/jo801580g
